# An iPSC-based model of 47,XYY Jacobs syndrome reveals a DNA methylation-independent transcriptional dysregulation shared with male X aneuploid cells

**DOI:** 10.1101/gr.279716.124

**Published:** 2025-07

**Authors:** Veronica Astro, Kelly Yojanna Cardona-Londoño, Lorena Viridiana Cortés-Medina, Rawan Alghamdi, Gustavo Ramírez-Calderón, Fotios Kefalas, Jair Dilmé-Capó, Santiago Radío, Antonio Adamo

**Affiliations:** 1 Biological and Environmental Science and Engineering Division, King Abdullah University of Science and Technology, Thuwal 23955-6900, Saudi Arabia;; 2 Sequentia Biotech SL, Barcelona 08005, Spain

## Abstract

Jacobs (JS) and Klinefelter (KS) syndromes, carrying 47,XYY and 47,XXY chromosomes, respectively, are the most prevalent sex-chromosome aneuploidies in males. JS and KS patients share several clinical features, including sterility, hormonal deficits, neurocognitive delay, and skeletal-muscle defects, although the penetrance of these traits in the two syndromes varies. Despite the high incidence, the molecular mechanisms underlying the clinical manifestations in sex aneuploid male patients are still elusive. In this study, we characterize the inaugural cohort of 47,XYY human induced pluripotent stem cells (iPSCs). We perform a comprehensive transcriptional analysis, including 47,XYY and 46,XY primary fibroblasts, iPSCs, and neural stem cells (NSCs), alongside a comparative analysis of 47,XYY and 47,XXY fibroblasts and iPSC transcriptomes. We reveal a transcriptional feedback mechanism tuning non-PAR X Chromosome gene (NPX) homologs in Y supernumerary cells, a phenomenon not detected in X aneuploid male iPSCs. By ectopically modulating the expression of selected NPY genes, we demonstrate a transcriptional link between the *UTY–KDM6A* gene pair. Furthermore, our analyses identify a shared transcriptomic signature between JS and KS, discernible already at the iPSC stage, with a notable enrichment for processes related to neurological functions. This transcriptomic convergence underscores potential commonalities in the molecular pathways underpinning the pathophysiology of male sex-chromosome aneuploidies. Finally, through genome-wide DNA methylation profiling of JS iPSCs, we demonstrate that a supernumerary Y Chromosome only minimally impacts the methylation status of 47,XYY cells at the pluripotent stage. Our work reveals critical transcriptional feedback mechanisms and shared gene expression signatures in male sex-chromosome aneuploidies.

Jacobs syndrome (JS) is the second most prevalent sex-chromosome aneuploidy in males, with a rate of 1:1000 newborns (Nielsen and Wohlert 1991; Abramsky and Chapple 1997; Davis et al. 2024). JS is characterized by an additional Y complement in males owing to paternal meiotic nondisjunction of the Y Chromosome, thus resulting in a 47,XYY karyotype. Despite the high incidence, a comprehensive understanding of the molecular mechanisms underlying the onset of the clinical features of JS patients remains elusive. Frequent phenotypic traits observed in JS patients include tall stature, macrocephaly, macrodontia, clinodactyly, scoliosis, increased testicular volume, and hypotonia (Bardsley et al. 2013; Berglund et al. 2020). Additionally, JS patients commonly exhibit neurological, cognitive, and behavioral deficits; an IQ score lower than siblings; and increased risks of hyperactivity and attention deficits (Ross et al. 2009; Bryant et al. 2012; Re and Birkhoff 2015; Ogutlu et al. 2018; Matsuzaki et al. 2019). Hormonal deficiencies, including hypogonadism, impaired testicular function, micropenis, and infertility, have also been reported (Bardsley et al. 2013; Rogol 2023). JS shares several neurocognitive characteristics with Klinefelter syndrome (KS), such as language disorders, tremors, anxiety, and intellectual disabilities (Ross et al. 2012). The shared phenotypic features may stem from the overexpression of genes within the pseudoautosomal region (PAR) 1 and non-PAR X-Y homologs. The PARs are located at the terminal ends of X and Y Chromosomes, spanning ∼2.6 Mb in Xp and Yp (PAR1) and ∼320 kbp in Xq and Yq (PAR2). Genes within PAR1 escape X inactivation and are expressed from the three sex chromosomes in 47,XYY (JS) and 47,XXY (KS) cells. The PAR1 region harbors 25 genes (Freije et al. 1992; Rappold 1993; Mangs and Morris 2007), whereas PAR2 contains only four genes, with two, *VAMP7* and *SPRY3*, predominantly inactive on the inactive X and on the Y Chromosomes (D'Esposito et al. 1996; Bonis et al. 2006). Another potential molecular mechanism underlying the shared neurological features of KS and JS involves the overdosage of expressed non-PAR Y genes (NPY) with homologs on the X Chromosome. Of particular relevance could be the overexpression of *NLGN4Y*, *UTY* (also known as *KDM6AL* or *KDM6C*), *ZFY*, and *DDX3Y* because the non-PAR X (NPX) homologs *NLGN4X, KDM6A* (also known as *UTX*), *ZFX*, and *DDX3X*, expressed from both X Chromosomes in KS males (Tukiainen et al. 2017), have been previously associated with autism spectrum disorder (ASD), intellectual deficits, developmental delay, and behavioral defects (Jamain et al. 2003; Laumonnier et al. 2004; Lederer et al. 2012; Zhu et al. 2013; Tang et al. 2021; Hoye et al. 2022; Gadek et al. 2023; Shepherdson et al. 2024). Despite the significant impact of sex-chromosome aneuploidies in humans and their high incidence among males, few models are available to study the effects of supernumerary Y and X Chromosomes on these conditions. Notably, no human induced pluripotent stem cell (hiPSC) models of JS have been described to date, highlighting the urgent need for viable in vitro models to untangle the impact of sex-chromosome aneuploidies during the earliest stages of human development.

In this study, we generated the inaugural cohort of JS iPSCs and iPSC-derived neural stem cells (NSCs) to assess the global transcriptional impact of Y-linked gene overdosage. Specifically, we compared primary 47,XYY fibroblasts to clonal iPSCs and NSCs to identify potential compensatory transcriptional mechanisms modulating the expression of NPX genes in response to NPY gene overexpression. Moreover, we coupled a cross-tissue transcriptomic and methylomic analysis of supernumerary Y Chromosomes to explore Y-mediated modulation of the autosomal genome. Finally, by combining JS and KS primary fibroblast and iPSC transcriptomes, we aimed to identify shared transcriptional signatures potentially linked to the common phenotypic outcome of the two diseases. Our work highlights the importance of implementing cellular models of clonal origin with minimized genomic background variability to dig into the molecular mechanisms dysregulated during the embryonic developmental stages of sex chromosomal aneuploidies.

## Results

### Generation of 47,XYY JS iPSCs and NSCs

**Figure 1.**
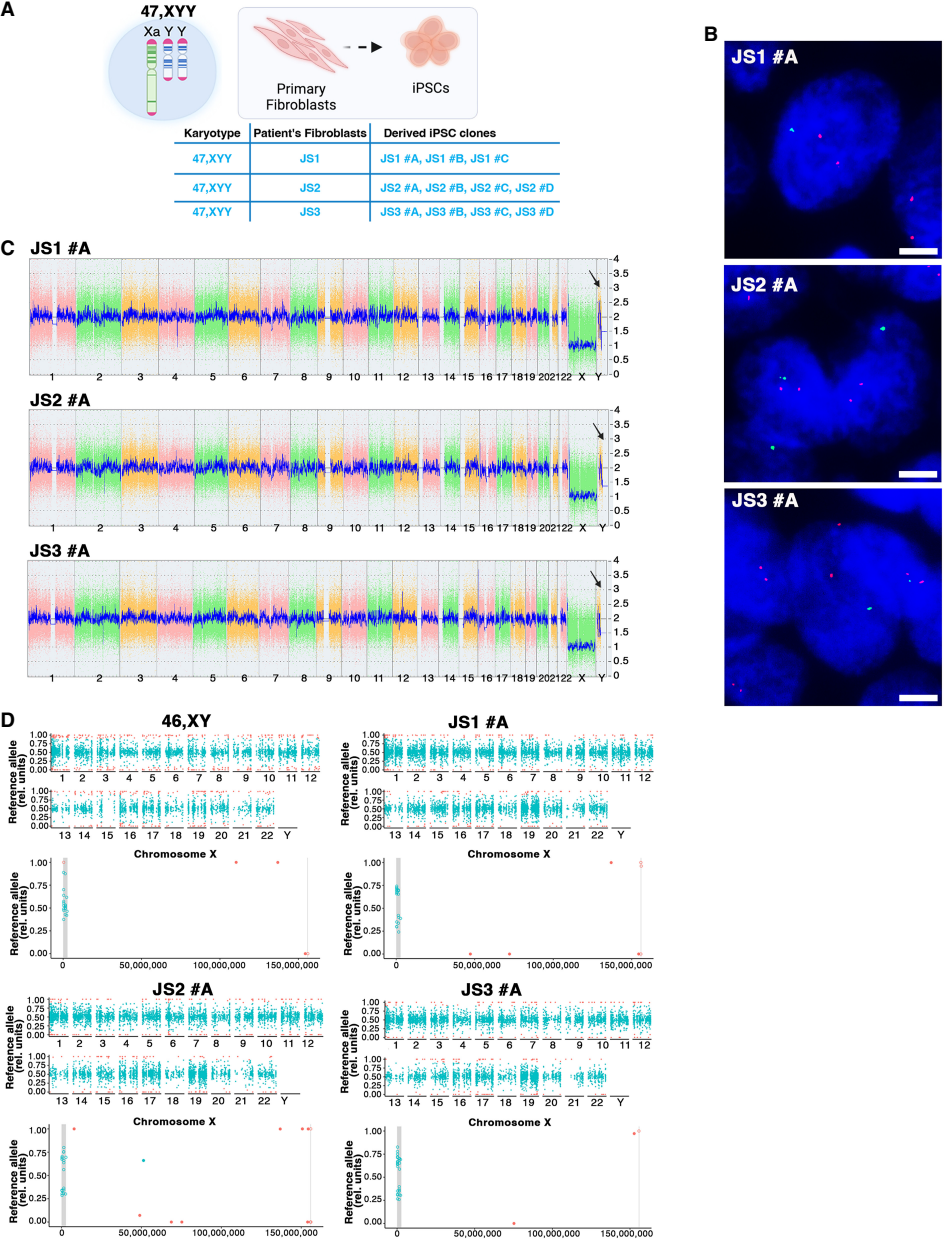
Characterization of 47,XYY JS iPSCs. (*A*) Schematic of the 11 47,XYY iPSC clones generated in this study. (*B*) Representative DNA-FISH images of X Chromosome (green) and Y Chromosome (red) in JS iPSCs. DNA was stained with DAPI (blue). Scale bar, 5 µm. (*C*) KaryoStat+ whole-genome view. The whole-genome view displays all somatic and sex chromosomes in one frame with a high-level copy number. The smooth signal plot (*right y*-axis) is the smoothing of the log_2_ ratios, which depict the signal intensities of probes on the microarray. The pink, green, and yellow colors indicate the raw signal for each chromosome probe, and the blue represents the normalized probe signal, which is used to identify copy numbers and aberrations. Black arrows indicate the gain of an additional Y Chromosome. (*D*) Scatter plot profiles of coupled WES analysis and allele-specific RNA-seq analysis performed on autosomal (*upper* panel) and sex chromosomes. The X Chromosomes are highlighted in the *lower* panel. Plots show the mono- (orange dots) or biallelic (light blue dots) gene expression status in two representative karyotypes (46,XY and 47,XYY). Gray rectangles indicate PAR1 and PAR2 regions, respectively. Solid dots indicate non-PAR genes; open dots show PAR genes. The scatter plot of iPSC clone JS2#A assigns a biallelic SNP to NUDT10. See the Methods section.

We selected a cohort of three nonmosaic 47,XYY JS patient fibroblasts through a bank repository (Fig. 1A; Supplemental Table S1). We cultured patient fibroblasts under identical conditions and validated the overexpression of the Y-linked genes *UTY*, *KDM5D*, and *ZFY* in JS versus 46,XY cells (Supplemental Fig. S1A). We used a virus-free, integration-free somatic cell reprogramming methodology to derive 47,XYY JS iPSCs (Supplemental Fig. S1B). We characterized three independent iPSC clones from each 47,XYY patient (Fig. 1A) and performed a short-tandem-repeat (STR) analysis to match the genetic profile of the parental fibroblasts and the derived iPSCs (Supplemental Table S2). Next, we assessed the sex-chromosome complement of the iPSCs with a dual approach. DNA-FISH assay confirmed the presence of one X and two Y Chromosomes in the nuclei of each 47,XYY iPSC clone (Fig. 1B; Supplemental Fig. S2). High-resolution KaryoStat assay confirmed a 47,XYY karyotype for all iPSCs generated in this study and excluded the presence of unbalanced chromosome rearrangement in all clones except for 1.2 and 2 Mb gain in clones JS1#B and JS2#B, respectively (Fig. 1C; Supplemental Fig. S3; Supplemental Data S1). Moreover, all iPSC lines exhibited canonical pluripotent morphology, expressing high pluripotency markers at the mRNA and protein levels (Supplemental Fig. S4), and displayed proper capability to differentiate into derivatives of the three germinal layers (Supplemental Figs. S5–S7). To model the consequences of Y aneuploidy during the earliest stages of human neurodevelopment, we differentiated 47,XYY and 46,XY iPSCs into NSCs, self-renewing multipotent intermediates from which the major cell types of the adult central nervous system arise (Gage and Temple 2013). JS and 46,XY iPSCs were differentiated into NSCs in vitro through a stepwise differentiation protocol (Supplemental Fig. S7A), leading to the robust induction of the neuroepithelial precursor marker *NES* and the neuronal marker *TUBB3* accompanied by the concomitant shutdown of the pluripotency gene *POU5F1* (also known as *OCT4*) (Supplemental Fig. S7B–D).

### Supernumerary Y Chromosome increases PAR and NPY gene expression levels

Genes within the human PARs on the Y and on the X are expressed from both sex chromosomes in male and female cells (Monteiro et al. 2021). We previously demonstrated that, in the presence of one or more supernumerary X Chromosomes, the expression of PAR1 genes mirrors the number of X Chromosomes (Astro et al. 2022). Thus, to ascertain whether a similar gene overexpression would be detected in supernumerary Y cells and to verify the biallelic expression profile of the PAR genes in JS, we coupled transcriptomic analysis to whole-exome sequencing (WES) and allele-specific expression (ASE) analysis in 47,XYY fibroblasts, iPSCs, and iPSC-derived NSCs. As expected, most autosomal genes displayed a biallelic expression profile. On the other hand, biallelically expressed SNPs were detected exclusively in the PAR1 region on the X and Y Chromosomes (Fig. 1D; Supplemental Fig. S8; Supplemental Data S2). Notably, given the bioinformatic convention of assigning PARs on the X while masking the Y Chromosome (Webster et al. 2019), every expressed PAR SNP is automatically mapped on Chromosome X. Moreover, because the supernumerary Y Chromosome in 47,XYY cells originates from a nondisjunction of sister chromatids of paternal origin (Robinson and Jacobs 1999), there are no biallelic expressed SNPs assigned to the Y Chromosome (Fig. 1D; Supplemental Fig. S8; Supplemental Data S2). Biallelic expressed SNPs mapping to the PAR1 region in JS samples were distributed around the values 30% and 60%, suggesting that the X and the two identical Y Chromosomes contribute similarly to the total expression level of the PAR1 transcripts. As expected, the expressed SNPs mapping to the PAR1 region in 46,XY controls clustered around 50% (Fig. 1D; Supplemental Fig. S8).

**Figure 2.**
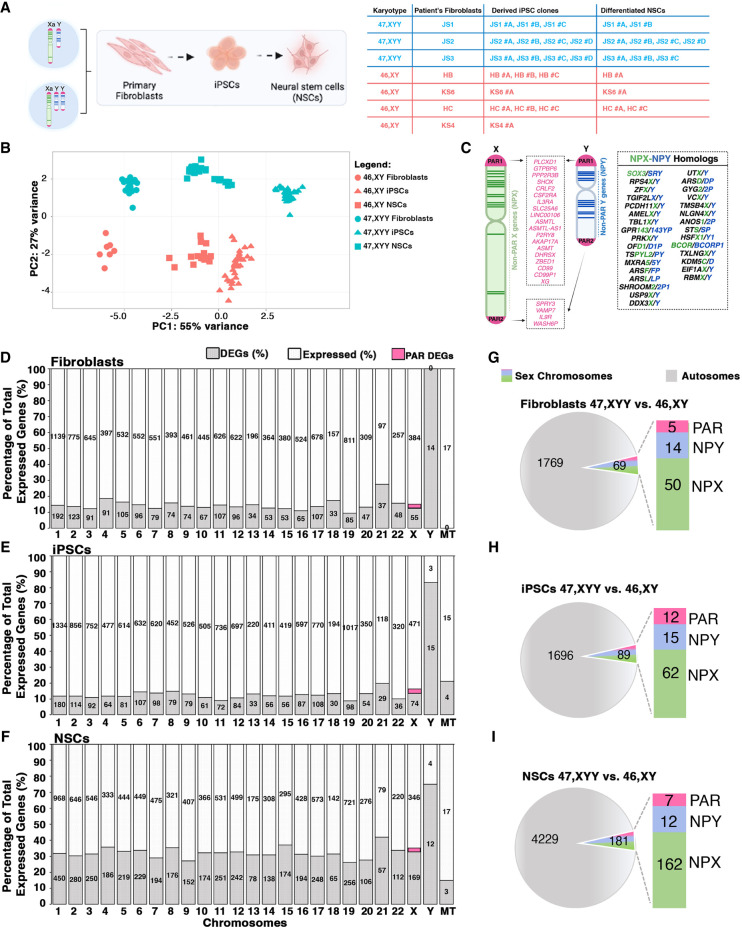
Transcriptional impact of Y Chromosome aneuploidy. (*A*) Illustration of the 47,XYY patient and control 46,XY fibroblasts and derived-iPSC clones used in the study. Three 47,XXY fibroblast lines have been reprogrammed to generate three independent 47,XYY iPSC clones for JS1 and four clones for JS2 and JS3. (HC) Healthy controls; (JS) Jacobs syndrome. The control 46,XY fibroblasts, and iPSCs have been described by Astro et al. (2022) and Astro and Adamo (2024). (*B*) PCA analysis of 46,XY (orange) and 47,XYY (blue) fibroblasts, iPSCs, and NCSs samples used in the study. (*C*) Schematic of human sex chromosomes listing the genes encoded from the X and Y Chromosome shared pseudoautosomal territories, PAR1 and PAR2, and from the divergent NPX and NPY regions. (*D*–*F*) Bar plots of the percentage of differential expressed genes (DEGs) over the total number of expressed genes in fibroblasts, iPSCs, and neural stem cells (NSCs). The specific number of DEGs and expressed genes are shown inside the bars for each chromosome. PAR DEGs are indicated in pink and assigned only to the X Chromosome. (MT) Mitochondrial genes. (*G*–*I*) Pie charts showing the number of autosomal and sex-chromosome DEGs in each cell type. The number of X- and Y-linked PAR, NPX, and NPY DEGs are indicated in the bars. Log_2_ fold change (Log_2_FC) > |0.58|, FDR < 0.05.

Next, we investigated the impact of the supernumerary Y Chromosome on the global transcriptome by profiling fibroblasts, iPSCs, and NSCs obtained from 47,XYY and 46,XY individuals (Fig. 2A). The unsupervised principal component analysis (PCA) of 47,XYY and 46,XY transcriptomes revealed that samples cluster according to cell type and karyotype (Fig. 2B). We detected 13,153, 14,900, and 13,989 expressed genes in fibroblasts, iPSCs, and NSCs, respectively (Supplemental Data S3). First, we analyzed the expression of Y- and X-linked genes residing within different chromosomal territories, the PAR, and the NPY and NPX regions. The Y Chromosome harbors a restricted number of genes, most of which have a homolog on the X (Fig. 2C). We counted 14, 18, and 16 NPY-expressed genes in fibroblasts, iPSCs, and NSCs, respectively (Fig. 2D–F; Supplemental Data S4). The expressed X-linked genes in fibroblasts, iPSCs, and NSCs are 439, 545, and 515, respectively. By studying the expression profile of PAR1 and PAR2 genes in the different cell types, we discovered that PAR gene expression is differentially regulated in a tissue-specific fashion (Supplemental Fig. S9).

**Figure 3.**
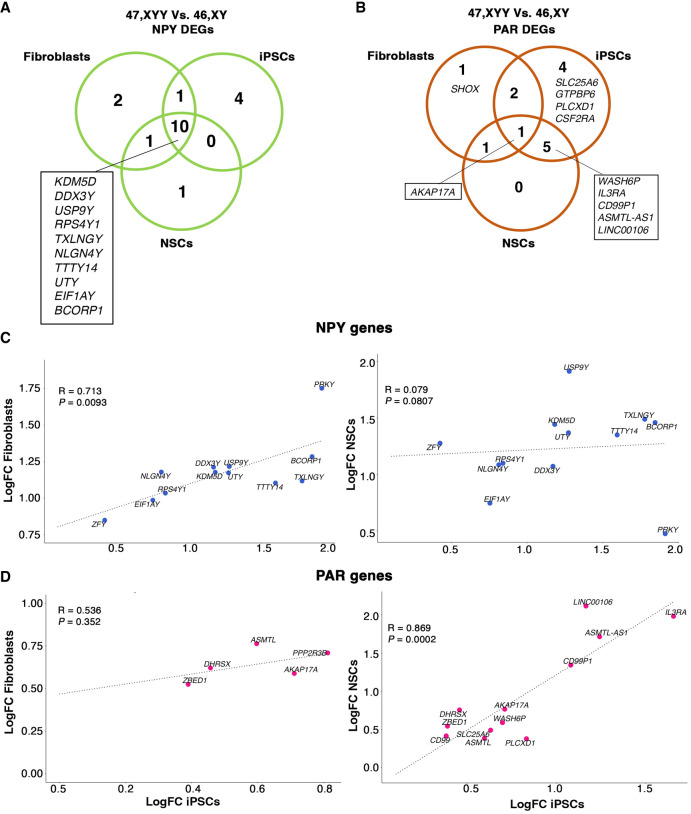
Transcriptomic profiling of sex-chromosome gene expression in 47,XYY cells. Venn diagrams of shared NPY DEGs (*A*) and PAR DEGs (*B*) in 47,XYY versus 46,XY fibroblasts, iPSCs, and NSCs. FDR < 0.05 and LogFC > |0.58|. (*C*,*D*) Scatter plot of the Log_2_FC comparison for NPY genes (*C*) and X- and Y-linked PARs (*D*) in fibroblasts versus iPSCs (*left*) and IPSCs versus NSCs (*right*). FDR < 0.01. NPY genes are indicated with blue dots. PAR genes are pink. The Pearson's correlation coefficient of the regression line (R) and *P-*values are shown.

The differential expression analysis (DEA) performed on 47,XYY and 46,XY karyotypes identified 1838, 1789, and 4413 differentially expressed genes (DEGs) in fibroblasts, iPSCs and NSCs, respectively (false-discovery rate [FDR] < 0.05 and Log_2_FC > |0.58|) (Fig. 2D–I; Supplemental Fig. S10; Supplemental Data S4, S5). Considering only the NPY-expressed genes, 14 (93%) in fibroblasts, 15 (83%) in iPSCs, and 12 (75%) in NSCs were identified as DEGs (Fig. 2D–I). Importantly, all NPY DEGs were upregulated in the three cell types except for *MXRA5Y*, a pseudogene of the X-linked gene *MXRA5*, which was downregulated in fibroblasts (Supplemental Fig. S11A–C). The observation that a Y-linked gene is downregulated despite its double dosage in JS could be the result of a transacting effect of sex-chromosome dosage on autosomes that subsequently affects other sex-chromosome-linked genes, a phenomenon previously described for X Chromosome aneuploidies (Raznahan et al. 2018; Astro et al. 2022). All PAR DEGs were upregulated and localized in the PAR1 region, with the exception of *XG*, downregulated in 47,XYY fibroblasts (Supplemental Fig. S9). *WASH6P* was the only PAR2 gene differentially expressed in iPSCs and NSCs (Supplemental Fig. S11D–F). Among the NPY DEGs, 10 are shared across all cell types, and all except *TTTY14* have NPX homologs (Fig. 3A). The genes *ENSG00000273906* and *ENSG00000288049* were identified as upregulated DEGs in 47,XYY-iPSCs, and NSCs, respectively (Supplemental Fig. S11A–C). Notably, there is no annotation of an X Chromosome homolog for these two genes. The identity of differentially expressed PAR genes varies across cell types (Fig. 3B; Supplemental Fig. S11D–F). Importantly, a correlation analysis of NPY and PAR DEGs comparing fibroblasts, iPSCs, and NSCs revealed a comparable magnitude of dysregulation of NPY and PAR genes across 47,XYY cells, except for NPY genes in NSCs against iPSCs (Fig. 3C,D).

### NPY gene overdosage leads to transcriptional modulation of NPX homologs

**Figure 4.**
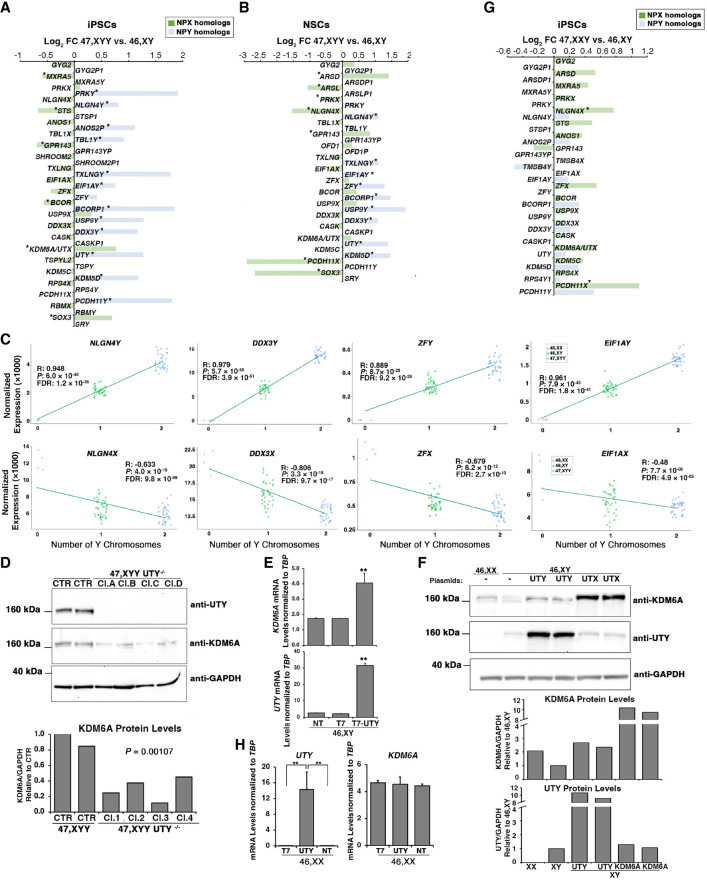
NPY genes modulate the expression of their homologs on the X Chromosome. (*A*,*B*) Log_2_FC of NPY (light blue) and NPX (green) expression in the contrast 47,XYY versus 46,XY in iPSCs (*A*) and NSCs (*B*). Expressed NPY–NPX genes are plotted. A single asterisk indicates DEGs with FDR < 0.05 and Log_2_FC > |0.58|. (*C*) Linear regression analysis of TMM normalized gene expression in iPSCs carrying zero (46,XX), one (46,XY), or two (47,XYY) Y Chromosomes. Pearson correlation coefficients (R), *P-*values, and FDR are indicated. (*Top*) NPY genes show a positive expression correlation with Y number. (*Bottom*) NPX homologs show an opposite correlation trend. (*D*, *top*) Western blot for UTY and KDM6A expression levels in 47,XYY *UTY*^−/−^ and *UTY*^+/+^ iPSCs. (*Bottom*) Quantification of KDM6A protein expression normalized on GAPDH levels in 47,XYY *UTY*^−/−^ and *UTY*^+/+^ iPSCs. Four independent *UTY*^−/−^ and two *UTY*^+/+^ (CTR.) clones (cl.) have been tested. The significance of the comparison between wild-type and knockout samples was calculated using the one-way ANOVA and pairwise comparison with post hoc Tukey HSD. (*E*,*F*) KDM6A and UTY overexpression in 46,XY H1 hESCs. (*E*) mRNA levels of *KDM6A* and *UTY* normalized to TATA-binding protein (*TBP*) in nontransfected (NT) cells or cells transfected with 3XT7 empty vector (T7) and 3XT7-UTY. Bars correspond to the average mRNA expression levels measured in triplicates from two independent transfections per sample. (*F*, *top*) Protein levels of KDM6A and UTY in cells overexpressing 3XT7-UTY (UTY) and 3XT7-KDM6A (KDM6A). (Lanes *1*,*2*) Basal protein levels of KDM6A and UTY in 46,XX and 46,XY cells. (Lanes *3*,*4*) Protein levels of KDM6A and UTY in 46,XY upon ectopic overexpression of UTY in two independent transfections. (Lanes *5*,*6*) Protein levels of KDM6A and UTY in 46,XY upon ectopic overexpression of KDM6A in two independent transfections. Untransfected female H9 and male H1 hESCs are used for antibody cross-checking. (*Bottom*) Quantification of the expression levels of KDM6A and UTY proteins normalized to GAPDH, relative to 46,XY levels. (*G*) Log_2_FC of NPX (green) and NPY (light blue) expression in the contrast 47,XXY versus 46,XY in iPSCs. Expressed NPY–NPX genes are plotted, DEGs with FDR < 0.05 and Log_2_FC > |0.58| are indicated with asterisks. (*H*) TaqMan assay for *KDM6A* and *UTY* mRNA in 46,XX H9 hESCs transfected with 3XT7 (T7) or 3XT7-UTY. Student's *t*-test (**) *P-*value < 0.005.

Next, we investigated whether the presence of a supernumerary Y Chromosome would lead to a transcriptional compensatory modulation of NPX genes. The DEA identified the X-linked genes whose expression positively or negatively changes in response to Y gene overexpression in fibroblasts, iPSCs, and NSCs (Supplemental Fig. S12). Notably, a subclass of NPX genes has an NPY homolog for which the degree of functional and structural similarities is mainly unexplored (Fig. 2C). Thus, we compared the expression of NPX and NPY homologs and identified *NLGN4X*, *EIF1AX*, *DDX3X*, and *TXLNGX* as genes significantly downregulated in 47,XYY iPSCs and NSCs. Conversely, their Y-homologs *NLGN4Y*, *EIF1AY*, *DDX3Y*, and *TXLNGY* were upregulated in both 47,XYY cell types (FDR < 0.01) (Fig. 4A,B). The NPX genes *ZFX*, *ANOS1*, and *BCOR* displayed an opposite expression trend compared with their NPY homologs in iPSCs but not in NSCs. The histone demethylase *KDM6A* and *USP9X* follow the same expression trend of their Y homologs (*UTY* and *USP9Y*) and are upregulated in iPSCs and NSCs. *KDM5C* is the only NPX gene consistently insensitive to increased Y homolog (*KDM5D*) expression in both cell types (Fig. 4A,B). Next, we assessed the correlation between the Y Chromosome dosage and the expression of sex-chromosome genes. To this aim, we generated a linear regression model with two (47,XYY), one (46,XY), or zero (46,XX) Y Chromosome complements by integrating the 46,XX iPSC transcriptomes previously obtained in our laboratory (Supplemental Data S6; Alowaysi et al. 2020; Astro et al. 2022, 2023). Our results demonstrate that a subset of NPX genes undergoes an inversely transcriptional regulation proportional to the number of Y Chromosomes, whereas others, such as *KDM6A*, are upregulated in JS cells (Fig. 4C). To test the hypothesis that NPY genes directly modulate the expression of their X homologs, we acutely knocked down *ZFY*, *NLGN4Y, DDX3Y*, and *UTY* in 47,XYY iPSCs using siRNAs. The knockdown efficiently reduced the expression of the targeted genes in three independent 47,XYY iPSCs (Supplemental Fig. S13A). Importantly, the *ZFX*, *NLGN4X*, and *DDX3X* expression was insensitive to *ZFY*, *NLGN4Y*, and *DDX3Y* knockdown. On the other hand, because of the high sequence homology between *KDM6A* and *UTY*, the siRNA targeting *UTY* partially led to *KDM6A* knockdown in female cells (Supplemental Fig. S13B). Given the epigenetic regulatory functions of the *KDM6A/UTY* homologs (Gažová et al. 2019) and the promising translational potential of the use of small molecules inhibiting these enzymes, we further investigated the potential transcriptional cross talk between *UTY* and *KDM6A* by employing a CRISPR-Cas9 strategy to knockout *UTY* in 47,XYY iPSCs (Supplemental Figs. S14, S15). *KDM6A* mRNAs and protein levels were significantly decreased in multiple 47,XYY *UTY*^−/−^ iPSC clones (Fig. 4D). To further validate these findings, we overexpressed UTY in 46,XY H1 human embryonic stem cells (hESCs). A 13-fold increase in UTY expression led to a onefold increase in KDM6A protein and mRNA levels (Fig. 4E,F). On the other hand, we did not detect a significant variation of UTY protein levels upon KDM6A overexpression in 46,XY H1 cells (Fig. 4F). Our results suggest a mechanism of transcriptional modulation of NPX homologs in JS iPSCs and NSCs, although an unequivocal regulation of single NPY–NPX gene pairs could only be detected for *UTY–KDM6A* in our model system.

Next, we investigated if, similarly to JS, the number of supernumerary X Chromosomes in KS iPSCs would correlate with the downregulation of the NPY genes. Integrating a data set of 47,XXY and 46,XY iPSCs transcriptomes (Astro et al. 2022, 2023), we discovered that none of the NPY genes were significantly downregulated in KS iPSCs (Fig. 4G). We concluded that NPY genes are mainly insensitive to X dosage. To further validate this evidence, we engineered 46,XY H1 hESCs to overexpress ZFX or KDM6A stably, and we did not observe significant changes in ZFY and UTY protein and mRNA levels (Supplemental Fig. S16). Recently, Rock et al. (2023) showed that the ectopic expression of *Uty* in mouse female hypothalamus does not alter *Kdm6a* expression. To evaluate the potentially different *KDM6A* transcriptional regulation in male and female cells, we overexpressed UTY in 46,XX H9 hESCs. We found that *KDM6A* is not sensitive to *UTY* dosage in female cells (Fig. 4H). Overall, our data indicate that a supernumerary Y Chromosome leads to the transcriptional dysregulation of several NPX genes but not vice versa, thus suggesting a unidirectional regulatory mechanism of a subset of Y genes on their X homologs.

### Y Chromosome aneuploidy leads to global transcriptional dysregulation

**Figure 5.**
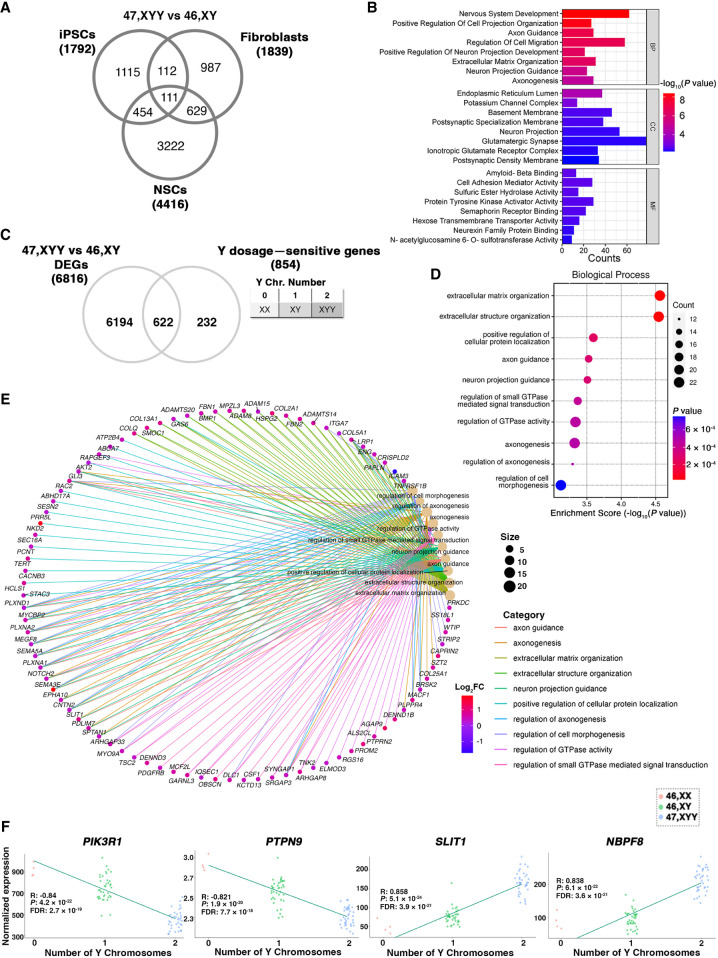
Autosomal response to Y Chromosome aneuploidy. (*A*) Venn diagram of shared DEGs comparing 47,XYY versus 46,XY, across three cell types. Log_2_FC > |0.58|, FDR < 0.05. (*B*) GO analysis of 1306 DEGs shared in at least two comparisons. (*C*) Venn diagram of DEGs in the 47,XYY versus 46,XY contrast and sensitive to Y Chromosome dosage. Dosage–sensitive genes are listed in Supplemental Data S6. FDR < 0.01 and Pearson's correlation value > |0.48|. (BP) Biological process; (CC) cellular component; (MF) molecular function. (*D*) Gene Ontology analysis on the DEGs responsive to Y Chromosome number showing significantly enriched biological processes. (*E*) Network analysis plot of genes belonging to enriched biological processes of the GO analysis shown in *D*. (*F*) Linear regression analysis of the TMM normalized gene expression in iPSCs carrying zero (46,XX), one (46,XY), or two (47,XYY) Y Chromosomes. The four top hits of autosomal genes with dosage sensitivity to the Y number are shown. Pearson's correlation coefficient (R), *P-*value, and FDR are indicated.

Next, we investigated the impact of Y aneuploidy on the global transcriptome of fibroblasts, iPSCs, and NSCs. We performed a Gene Ontology (GO) analysis, selecting genes commonly dysregulated in at least two cell types (Fig. 5A). We identified enrichment for terms related to neuron system development, axon guidance, cell migration, and neuron projection (Fig. 5B), all processes particularly significant for JS phenotypic features. We narrowed our analysis to iPSC DEGs responsive to Y Chromosome dosage. To this end, we built a correlation model using iPSCs with zero (46,XX), one (46,XY), and two (47,XYY) Y Chromosomes, and we detected 854 genes sensitive to Y-dosage. Additionally, we crossed Y-dosage-sensitive genes with the DEGs in iPSCs (47,XYY vs. 46,XY) and identified 622 DEGs (FDR < 0.01) whose expression changes proportionally to the number of Y Chromosomes (Fig. 5C). The GO analysis on these DEGs confirmed that genes associated with neuronal morphogenesis and function are dysregulated at the pluripotent stage and are sensitive to Y Chromosome dosage (Fig. 5D,E). Among the Y-sensitive autosomal genes, *NBPF8* and *SLIT1* had the highest positive correlation, whereas *PIK3R1* and *PTPN9* had the highest negative correlation with the number of Ys (Fig. 5F).

### The presence of an extra Y Chromosome results in subtle changes in DNA methylation

To understand the impact of an extra Y Chromosome on global DNA methylation, we performed a genome-wide methylation analysis (GWMA) using reduced representation bisulfite (RRBS) sequencing (RRBS-seq) on primary fibroblasts and iPSCs. The PCA of the general distribution of CpGs in 47,XYY and 46,XY demonstrated that the global methylation is primarily influenced by cell type rather than karyotype (Supplemental Fig. S17A). We found that the number of significant differentially methylated CpGs in fibroblasts is higher than in iPSCs (Supplemental Fig. S18A,B). Next, we assessed the methylation status of the Y Chromosome in the two cell types. Primary fibroblasts displayed a heterogeneous pattern of CpG methylation, potentially ascribable to the nonclonal nature of this cell population (Supplemental Fig. S18C; Wagner et al. 2014). On the other hand, methylated CpGs in clonal iPSC lines are significantly more homogenous (Supplemental Fig. S18D). Finally, we analyzed the differentially methylated regions (DMRs) comparing 47,XYY and 46,XY cells and identified 4197 and 63 DMRs in fibroblasts and iPSCs, respectively (Supplemental Fig. S17B; Supplemental Data S7). Overall, the small number of DMRs identified in 47,XYY versus 46,XY iPSCs indicates that the supernumerary Y minimally affects the global DNA methylation landscape at this developmental stage. Coherently, the few identified DMRs in iPSCs do not correlate with differential gene expression (Supplemental Fig. S19A). On the other hand, we observed a significant hypermethylation trend in 47,XYY primary fibroblasts (Supplemental Fig. S18E). In these cells, the DMRs in autosomal chromosomes represent ∼ 97% of the total detected DMRs (Supplemental Fig. S17B) and are uniformly distributed among intergenic, promoter, and gene body genomic regions (Supplemental Fig. S18F). The DMRs detected in sex chromosomes (3%) are also predominantly hypermethylated and mostly intergenic. We identified only a few hypermethylated DMRs (eight) on the Y Chromosome (Supplemental Fig. S18G). Next, we correlated the DEGs detected in the 47,XYY versus 46,XY contrast in fibroblasts with hyper- and hypomethylated DMRs in promoters and gene bodies. Notably, we found a similar distribution of upregulated and downregulated genes over DMRs regardless of their genomic location, possibly ascribable to primary cell heterogeneity or the small sample size (Supplemental Fig. S19B; Supplemental Data S8).

### Identification of a shared transcriptional signature in Y and X Chromosome aneuploidies

**Figure 6.**
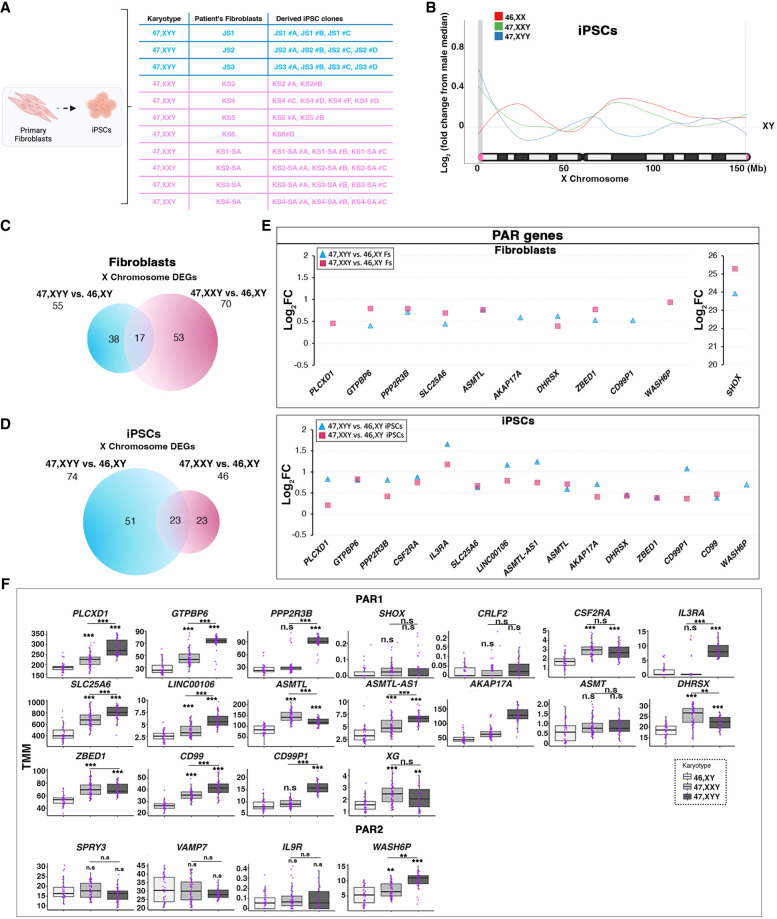
Y and X Chromosome aneuploidies contribute differently to PAR gene overexpression. (*A*) Schematic of 46,XY, 47,XYY, and 47,XXY fibroblasts and iPSC cohort analyzed in the present study. Fibroblasts from four males, three JS, and eight KS patients have been used to derive eight 46,XY, nine 47,XYY, and 20 47,XXY independent iPSC clones. (*B*) Moving average line plot (LOESS fit, span 0.45) along the X Chromosome showing the Log_2_FC from control 46,XY iPSCs. Gray vertical boxes indicate PAR1 and PAR2 regions. The gray horizontal line represents no theoretical deviations from control 46,XY samples. (*C*,*D*) Venn diagram showing overlapping X-linked DEGs in the 47,XYY versus 46,XY (blue circle) and 47,XXY versus 46,XY (pink circle) contrasts in fibroblasts (*C*) and iPSCs (*D*). FDR < 0.05 and Log_2_FC > |0.58|. (*E*) Log_2_FC of detected PAR genes in 47,XYY and 47,XXY versus controls, fibroblasts (*top*), and iPSCs (*bottom*). (*F*) Box plot of PAR gene expression in 46,XY, 47,XXY, and 47,XYY iPSCs. (TMM) Trimmed mean of M-values. Each purple dot represents an independent RNA sample. The significance of the comparison between 47,XXY and 47,XYY versus 46,XY was calculated using the one-way ANOVA and pairwise comparison with post hoc Tukey HSD. (**) *P*-value < 0.01; (***) *P*-value < 0.001; (ns) not significant.

Given JS and KS patients’ highly similar phenotypic features, we investigated if a shared transcriptional impact of supernumerary Y or X Chromosomes in JS and KS fibroblasts and iPSCs could be identified. We used a cohort of eight primary KS patients’ fibroblasts and 18 47,XXY iPSC clones derived in our previous studies (Astro et al. 2022, 2023) to perform a comparative analysis on 47,XYY and 47,XXY cells (Fig. 6A). First, we determined the impact of a supernumerary X or Y Chromosome on X-linked genes by plotting the X-linked Log_2_FC moving average in 46,XX, 47,XXY, and 47,XYY normalized on 46,XY iPSCs (Fig. 6B). We found that 47,XYY and 47,XXY have a similar density trend of increased gene expression along the terminal Xp arm, corresponding to the PAR1 region. Our results highlight that JS iPSCs express PAR1 genes at a higher level than KS cells. Next, we evaluated if the non-PAR transcriptional differences observed at the Xp arm between JS and KS are ascribable to NPX genes with an NPY homolog. To visually exacerbate transcriptional differences in the chromosomal territory proximal to PAR1, we built an alternative X-linked Log_2_FC moving average excluding PAR1 genes (Supplemental Fig. S20). Our results demonstrate that the transcriptional upregulation in the Xp arm in JS and KS is mainly restricted to the PAR1 region, whereas the expression of NPX genes proximal to PAR1 in JS is predominantly downregulated compared with KS and 46,XX cells.

**Figure 7.**
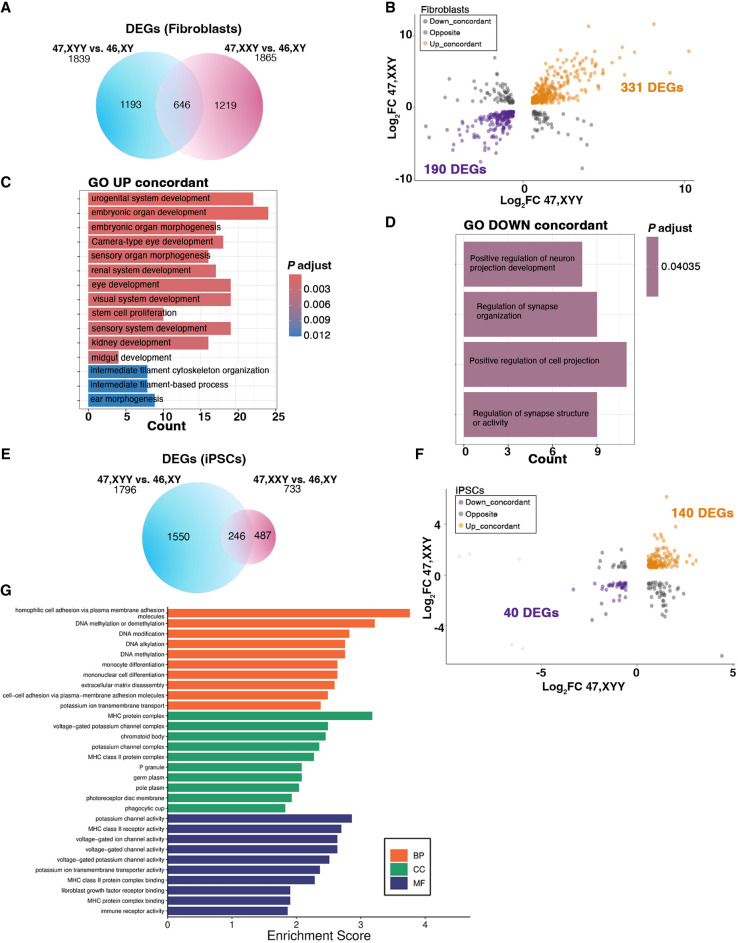
The shared dysregulated genes in X and Y aneuploidies define the transcriptomic signature of sex aneuploidies. (*A*) Venn diagram showing overlapping DEGs in the 47,XYY versus 46,XY (blue circle) and 47,XXY versus 46,XY (pink circle) contrasts in fibroblasts. FDR < 0.05 and LogFC > |0.58|. (*B*) Genome-wide interaction analysis showing the number of DEGs that display an opposite or concordant dysregulation trend in the two contrasts in fibroblasts. For the complete gene list, see Supplemental Data S9. (*C*,*D*) Gene Ontology enrichment analysis on concordantly upregulated (*C*) or downregulated (*D*) DEGs in the KS and JS versus 46,XY fibroblasts. (*E*) Venn diagram showing overlapping DEGs in the 47,XYY versus 46,XY (blue circle) and 47,XXY versus 46,XY (pink circle) contrasts in iPSCs. (*F*) Genome-wide interaction analysis on DEGs with opposite or concordant dysregulation in the two contrasts in iPSCs. For the complete gene list, see Supplemental Data S9. (*G*) Gene Ontology enrichment analysis on the shared concordant DEGs in the KS and JS versus 46,XY fibroblasts. FDR < 0.05 and Log_2_FC > |0.58|. (BP) Biological processes; (MF) Molecular functions; (CC) cellular components.

Moreover, although 47,XXY and 46,XX have a similar expression density on the Xq arm, indicating that the second X leads to a similar transcriptional contribution in female and male cells, the additional Y Chromosome leads to a mild downregulation along the X Chromosome, thus diverging from both KS and female cells (Fig. 6B). Next, we compared the 47,XYY versus 46,XY and the 47,XXY versus 46,XY contrasts in fibroblasts and iPSCs and identified 30% and 31%, respectively, commonly dysregulated X-linked DEGs (Fig. 6C,D; Supplemental Data S9). Importantly, half of the shared DEGs are PAR genes, and they are all upregulated versus 46,XY cells (Fig. 6E). In iPSCs, the 47,XYY versus 46,XY contrast displayed a higher fold change increase of the shared PAR DEGs compared with the 47,XXY versus 46,XY contrast, thus corroborating the hypothesis that supernumerary Y and X Chromosomes contribute differently to PAR gene expression levels (Fig. 6E,F). However, although clonal cell populations such as iPSCs allow a more quantitative evaluation of the transcriptional contribution of an extra Y or X Chromosome, primary cells are more subjected to confounding effects owing to cell heterogeneity (Supplemental Fig. S21). Finally, we performed an interaction analysis to identify the dysregulation trends among the shared DEGs in 47,XYY and 47,XXY. In fibroblasts, out of 646 common DEGs, 80% are concordantly upregulated (331) or downregulated (190) (Fig. 7A,B; Supplemental Data S9). The GO analysis performed on the concordant DEGs highlighted terms related to KS and JS clinical features, such as urogenital system development, neuron projection development, and synapse organization (Fig. 7C,D). In iPSCs, out of 246 common DEGs, 74% are concordantly upregulated (140) or downregulated (40) (Fig. 7E,F) and are significantly enriched for multiple GO terms related to voltage-gated ion channel activities and immune response (Fig. 7G). Collectively, our results suggest that a shared transcriptomic signature associated with sex-chromosome aneuploidies in males could constitute the molecular landscape leading to the common phenotypic features in JS and KS patients.

## Discussion

Our study delves into the intricate biological implications of an additional Y Chromosome in JS, drawing parallels to X aneuploidies in males. Through the successful generation and meticulous characterization of 47,XYY-iPSCs from nonmosaic JS patient fibroblasts, we inaugurated a pivotal understanding of the effects of Y Chromosome aneuploidy in human pluripotency and its subsequent impact during differentiation toward disease-relevant lineages. Our methodology hinges upon an mRNA-mediated reprogramming approach, a technique validated for its efficacy in mitigating reprogramming-induced spontaneous sex chromosomal loss, inter-clone heterogeneity, and insertional mutagenesis events (Warren et al. 2010; Pasi et al. 2011; Adamo et al. 2015; Fiacco et al. 2021; Astro et al. 2022; Astro and Adamo 2024). This approach ensured the establishment of a homogeneous cellular platform, thereby furnishing a conducive milieu for in vitro studies.

Prior investigations into the transcriptional impact of Y and X Chromosome aneuploidies predominantly relied on primary patient cells or immortalized lymphoblastoid cell lines (Viana et al. 2014; Zitzmann et al. 2015; Belling et al. 2017; Raznahan et al. 2018; Skakkebæk et al. 2018; Winge et al. 2018; Zhang et al. 2020a; Roman et al. 2023, 2024). Although these studies have been instrumental in unraveling common cellular dysregulations associated with these conditions across diverse patient cohorts, they are encumbered by intrinsic heterogeneity stemming from different genetic backgrounds, ethnicity, age, environmental factors, and potential cryptic mosaicism across various tissues. Conversely, cellular models of clonal origin, such as iPSCs, are limited by a smaller cohort size but allow the establishment of an in vitro platform with minimized variability. Additionally, iPSCs enable modeling the cellular and molecular consequences of sex-chromosome aneuploidies during the earliest stages of embryonic development and the systematic investigation of the impact exerted by the additional copy of Y- and X-linked genes on the global transcriptome with tissue-specific resolution.

Leveraging our refined cellular model, we conducted a comprehensive transcriptional analysis encompassing JS fibroblasts, iPSCs, and NSCs juxtaposed with KS cells. This endeavor unveiled intriguing differences in the expression of most PAR1 genes, observing higher expression levels in 47,XYY compared to 47,XXY. This finding prompts a deeper investigation into whether the transcriptional contribution of the PAR gene copy on the inactive X is equivalent to the one on the Y or whether it is negatively modulated by the surrounding heterochromatic environment in female and KS cells, as previously suggested (Brown et al. 1997; Carrel and Willard 2005; Yang et al. 2010; Berletch et al. 2011). Our work demonstrated that the expression of several NPX homologs in male cells is sensitive to the expression levels of NPY genes, a phenomenon not detected in X aneuploid male iPSCs. Although we show that *UTY* levels impact those of *KDM6A* in male iPSCs, whether this regulation is exerted through direct regulation of the *KDM6A* promoter or indirectly through the regulation of a transcription factor that, in turn, regulates *KDM6A* remains to be elucidated. A hint toward an indirect regulation is the observation that in response to the ectopic overexpression of UTY in male cells, KDM6A is not stoichiometrically upregulated at mRNA and protein levels. Future studies will elucidate the potential direct or indirect cross talk of NPY–NPX homologs whose converging or diverging function in defining sex-biased traits has been recently debated (Wilson 2021; DeCasien et al. 2025).

Furthermore, our analyses identified a shared transcriptomic signature between JS and KS, discernible already at the iPSC stage, with significant enrichment for processes related to neurological functions. However, although this transcriptomic convergence underscores potential commonalities in the molecular pathways underpinning the pathophysiology of male sex-chromosome aneuploidies, whether this common transcriptional dysregulation is conserved during neuronal differentiation remains to be addressed. These results are particularly relevant considering that minimal DNA methylation differences exist between 47,XYY and 46,XY cells. Although the well-established phenomenon of DNA methylation spreading on the supernumerary X Chromosome in KS has been linked to a global transcriptional impact in primary cells (Miao et al. 2023; Viuff et al. 2023), our findings in JS iPSCs could challenge this notion. The identification of highly similar patterns of DEGs in JS and KS prompts us to hypothesize that, in X Chromosome male aneuploidies, the DNA methylation of the supernumerary X Chromosome could play a marginal role in regulating the global transcriptome at the pluripotent stage.

## Methods

### Fibroblast reprogramming into iPSCs

Fibroblasts were obtained from the NIGMS Human Genetic Cell Repository at the Coriell Institute for Medical Research (Supplemental Table S1). The reprogramming was performed as described by Astro and Adamo (2024). Additional technical details are provided in Supplemental Materials.

### Human stem cell culture

The established hiPSC lines were cultured on hESC-qualified Matrigel (Corning) coated 6-well plates in E8 media and passaged with Versene in E8 supplemented with 5 µM rock inhibitor (Y-27632). Human iPSC lines were incubated at 37°C in 5% CO_2_ and 5% O_2_. The 46,XY H1 (WA01) and 46,XX H9 (WA09) hESCs used in this study were obtained from the WiCell biobank. We performed KaryoStat analyses and DNA fluorescence in situ hybridization (DNA-FISH) to detect chromosomal abnormalities in iPSC clones and fibroblasts’ donors, whereas genomic profiling matching of fibroblast's donors and iPSCs was performed using X Chromosome STR (Supplemental Materials; Supplemental Table S3). Additional details for pluripotency validation, plasmid generation, stem cell transfection and genome editing are provided in the Supplemental Materials and Supplemental Table S4.

### Differentiation of iPSCs into NSCs

The differentiation of iPSCs into NSCs was established using the PSC neural induction kit from Thermo Fisher Scientific. Briefly, iPSCs were detached when they reached 70%–80% confluency in small clumps using Versene and seeded at a dilution 1:6–1:8 on Matrigel-coated dishes in the presence of E8+10 µM Y-27632. Twenty-four hours after seeding, the neural induction media (neurobasal medium and neural induction supplement) was added and used for 7 days. On day 7 of neural induction, NSCs that reached P0 were harvested using Accutase, seeded at 1.0 × 10^6^ cells on Matrigel-coated 6-well plates, and cultured in neural expansion media (50% neurobasal medium, 50% advanced DMEM/F12 media, and neural induction supplement) for ∼5 days, until they reached 90% confluency. NSCs have been further expanded in neural expansion media and collected at P2 for RNA extraction and immunostaining.

### Bulk RNA-seq library preparation and sequencing

RNA extraction and qPCR analyses are described in Supplemental Materials and Supplemental Table S4. RNA libraries were generated using the human mRNA TruSeq stranded library preparation kit from Illumina and profiled using NovaSeq 6000 and NovaSeq XPlus systems with 150 bp paired-end sequencing method. An average of 30 million reads were obtained for each sample. Samples with fewer than 16 million input reads and <75% assigned reads were removed from the analysis (Supplemental Data S10, S11).

### RNA-seq data processing and analysis

To assess the quality of the raw RNA-seq data, we used the FastQC tool (http://www.bioinformatics.babraham.ac.uk/projects/fastqc/). This was followed by trimming adapter sequences, filtering out low-quality bases with a minimum quality score of 25, and discarding reads shorter than 35 bases with BBDuk (BBmap suite v37.62). Then, we performed a quality reassessment of the trimming step with FastQC. The preprocessed high-quality reads were then aligned to the GRCh38.101 release of the Ensembl human reference genome using the STAR software v. 2.7.10 (Dobin et al. 2013). To maintain high precision in the alignment process, we permitted a maximum of three mismatches per read (‐‐outFilterMismatchNmax 3) and limited the mismatch ratio to no more than 10% of the read length (‐‐outFilterMismatchNoverLmax 0.1). The alignment quality was further ensured by adopting at least a 66% alignment score of the maximum possible score based on the read length (‐‐outFilterScoreMinOverLread 0.66), effectively filtering out lower-quality alignments. We used featureCounts (Subread suite v. 2.0.2) (Liao et al. 2014) to quantify gene expression levels. To address potential batch effects, we used ComBat-seq (Zhang et al. 2020b). To exclude potential inaccuracies in mapping RNA-seq reads from Y and X Chromosome genes and verify that multimapped reads (excluded by the mapping algorithm) do not significantly bias the number of univocally assigned reads, we used a dual approach: a simulation of Y Chromosome transcriptome RNA-seq reads and multimapping assessment on iPSC transcriptomic data (Supplemental Data S12; Supplemental Materials).

We divided the adjusted count matrix per cell type to independently perform the gene filtering and DEA. Genes with low expression were removed from the analysis using the HTS Filter package (Rau et al. 2013). Differential gene expression analysis was conducted via the DESeq2 package (Love et al. 2014) in R version 4.3.1 (R Core Team 2023). We applied a FDR cutoff of less than 0.05 and a |Log_2_FC| > 0.58 threshold to consider a gene differentially expressed. When specified in the figure legends, an FDR < 0.01 threshold was applied for specific analysis and plots. The RNA-seq data from our previous studies that we have reanalyzed in this study are publicly available at the NCBI Gene Expression Omnibus (GEO; https://www.ncbi.nlm.nih.gov/geo/) under accession numbers GSE152001 and GSE220268. Additional methods for Y Chromosome transcriptome simulation and multimapping assessment on RNA-seq data, WES, and ASE analysis are described in the Supplemental Materials.

### Bootstrap sampling

To investigate whether the large number of DEGs identified in the 47,XYY versus 46,XY NSCs contrast was influenced by clonal variation rather than genuine karyotype differences, we implemented a bootstrap approach. This method was designed to assess the stability of identified DEGs across different combinations of clonal samples for iPSCs and NSCs. We used the iPSC and NSC filtered count matrices to perform 50 bootstrap iterations, each time selecting a random clone from each patient. For each iteration, a clone was randomly selected from each patient's pool of clones, and all other clones from the same patient were excluded. The resulting count matrix was normalized, and a DEA was performed using DESeq2. The model design was set to ∼group, and the contrast 47,XYY versus 46,XY.

DEGs were identified with a FDR threshold of 0.05 and a log_2_FC cutoff > ± 0.58. Finally, we aggregated the results across all bootstrap iterations, calculating the percentage of iterations in which each gene was identified as a DEG (Supplemental Data S5).

### Identification of genes with dosage sensitivity to Y Chromosome copy number

We used the count matrix for the iPSCs samples to perform the correlation analysis. We applied batch correction with ComBat-seq, removed low-expressed genes with the HTS Filter package, calculated normalized values using DESeq2, and then performed a Pearson's correlation for every expressed gene (Y ∼ E, where Y is the number of Y Chromosomes, and E is the normalized expression).

### Moving expression average along the X Chromosome

Healthy male (XY) samples (n = 34) were used as a reference to calculate the median FPKM level of each gene (male median) after removing nonexpressed genes as previously described (Bar et al. 2019). Additionally, n = 6 female, n = 24 KS, and n = 37 JS iPSC samples were plotted. Next, for every sample, the FPKM level of each gene was divided by the male median. These fold changes were then plotted in a moving average plot using a LOESS fit, adjusting the span to 0.45 and determining the sliding window size as a proportion of observations in each local regression. To generate the alternative X-linked Log_2_FC moving average, we used the same steps but excluded the PAR1 genes:

(1) $$\begin{array}{rl} \mathrm{MM}\left(\text{Male median}\right) & =\mathrm{Median}\left(\text{FPKM in reference XY samples}\right)\\ & \text{ of a specific gene}\\ \mathrm{FC}\left(\text{Fold Change}\right) & =\text{FPKM in each sample/MM of a }\\ & \mathrm{specific}\;\mathrm{gene} \end{array}(1)$$

### RRBS library preparation and sequencing

The gDNA was extracted using the DNeasy blood and tissue genomic DNA extraction kit from Qiagen. Beijing Novogene Bioinformatics Technology generated the DNA libraries. Then, the gDNA was sequenced using Illumina NovaSeq 6000 or NovaseqX 2 × 150 bp.

### RRBS data analysis

The quality of raw data was assessed using FastQC. We then proceeded with adapter trimming C using Trim Galore! v.0.6.6 to prepare the DNA methylation data. After a quality reassessment using FastQC, the high-quality reads were aligned to the GRCh38.105 release of the Ensembl human reference genome using Bismark v.0.23.1. We detected about 5.2 million CpGs in iPSC samples, 5.4 million in NSC samples, and 5.6 million in fibroblast samples. CpG site methylation extraction was conducted with MethylDackel v.0.6.1. Then, differential methylation analysis was performed with the DSS package in R (Feng et al. 2014). We defined DMRs by a minimum length of 50 bp and a minimum of three CpGs. DMRs in a distance within 50 bp of one another were merged. We established a significance threshold for loci at 1 × 10^−5^, requiring that DMRs contain at least 50% differentially methylated CpGs throughout their entire length after the merging.

## Data access

The RNA-seq and RRBS-seq data generated in this study have been submitted to the NCBI Gene Expression Omnibus (GEO; https://www.ncbi.nlm.nih.gov/geo/) under accession numbers GSE264395 and GSE264396, respectively.

## Supplemental Material

Supplement 1

Supplement 2

Supplement 3

Supplement 4

Supplement 5

Supplement 6

Supplement 7

Supplement 8

Supplement 9

Supplement 10

Supplement 11

Supplement 12

Supplement 13

Supplement 14

Supplement 15

Supplement 16

Supplement 17

Supplement 18
