# Supplementary material for: An iPSC-based model of 47,XYY Jacobs syndrome reveals a DNA methylation-independent transcriptional dysregulation shared with male X aneuploid cells
Source: Genome Res. 2025 Jul;35(7):1503–17. doi: 10.1101/gr.279716.124 (PMC12212075; doi:10.1101/gr.279716.124)
Supplement: Supplement 1 [file Supplemental_Materials_R3.docx]

**SUPPLEMENTAL METHODS**

**Fibroblast reprogramming into iPSCs**

Fibroblasts were obtained from the NIGMS Human Genetic Cell Repository at the Coriell Institute for Medical Research (**Supplemental Table S1**), cultured, and expanded for at least three passages in high glucose DMEM supplemented with 15% FBS, 1% non-essential amino acids, and 1% penicillin-streptomycin before cell reprogramming. About 5 × 10^4^ –10 × 10^4^ cells/well were seeded on iMatrix-coated 6-well plates 24 h before reprogramming. The reprogramming was performed as described in(Astro and Adamo 2024).

**Human stem cell culture and transfection**

The established hiPSC lines were cultured on hESC-qualified Matrigel (Corning) coated 6-well plates in E8 media and passaged with Versene in E8 supplemented with 5 μM Rock Inhibitor (Y-27632). Human iPSC lines were incubated at 37°C in 5% CO_2_ and 5% O_2_. The 46,XY H1 (WA01) and 46,XX H9 (WA09) hESCs used in this study were obtained from the WiCell biobank. hESCs were cultured on vitronectin-coated (VTN, Thermo Fisher Scientific) and E8 Medium, incubated at 37°C, 5% CO_2_, and passaged using Accutase (Thermo Fisher Scientific) and 5 μM Rock Inhibitor (Y-27632).

For NPY gene silencing, 47,XYY iPSCs have been transfected with selected siRNAs 50nM (**Supplemental Table S4**) using RNAiMAX Lipofectamine (Thermo Scientific). Cells were incubated in E8 media for two days after transfection before collection for RNA extraction. The acute transfection of 46,XY H1 and 46,XX H9 hESCs was performed with 4.5 ug of pTP6-3XT7 empty vector (control), pTP6-3XT7-KDM6A or pTP6-3XT7-UTY.

**KaryoStat analyses and DNA Fluorescence *in situ* hybridization (DNA-FISH)**

A KaryoStat assay was used to accurately detect chromosomal abnormalities in the three iPSC clones obtained from the three fibroblasts’ donors. The assay, including arrays, reagents, and data analysis, was performed by Thermo Fisher Scientific.

The DNA Fluorescence in situ hybridization (DNA-FISH) was performed to validate further the Y copy number in the 47,XYY iPSC clones. iPSCs were cultured on Matrigel-coated chambered glass slides (Thermo Fisher, 177402) and fixed in a fixative solution containing 3:1 Methanol: Acetic Acid. After the Pepsin digestion at 37°C for 2-4 minutes, the slides were incubated in formaldehyde for 10 minutes and dehydrated in an ethanol series, 70%, 85%, and 100%, for 2 minutes each. The slides were then denatured in the presence of X and Y Chromosome probes (CytoCell, LPE 0XYc / LPE 0XYq) on a heat block at 75C for 5 minutes and hybridized at 37°C overnight in a humidity chamber. After two washes in 1X SCC solution (Thermo Fisher, AM9763), at 37C and 45C, the slides were incubated in the presence of 4x SSC, 0.1% Tween 20 for 1 minute at room temperature, followed by an ethanol series dehydration, 70%, 85%, and 100%, for 3 minutes each. The slides were mounted with ProLong Glass antifade Mounting solution with DAPI (Thermo Fisher Scientific, P36931). DNA-FISH images were acquired as Z-stacks, processed by maximum intensity projections, and computer cleared using a DMi8 THUNDER Imager (Leica) using a 1.4 NA/100X oil immersion objective (Leica) and LAS X software. The probes used for DNA-FISH are listed in **Supplemental Table S3**.

**X Chromosome short tandem repeat (STR)**

The STR analysis was performed on genomic DNA using a GlobalFiler kit (Thermo Fisher Scientific) following the manufacturer’s recommendations. A total of 24 loci were amplified, including 21 autosomal STR loci and three gender-linked loci. PCR amplicons with the internal size standard GeneScan 600 LIZ Size Standard v2.0 were loaded and resolved on SeqStudio Genetic Analyzer (Thermo Fisher Scientific). Data were analyzed with GeneMapper ID-X software v1.6 (Applied Biosystems).

**Plasmid generation, stable transfection, and CRISPR-Cas9 mediated genome editing of *UTY* gene.**

The pTP6-3XFlag-KDM6A was obtained through EcoRI digestion and subcloning into the pTP6 vector(Pratt et al. 2000) of the 3XFlag-KDM6A sequence optimized for human expression (purchased from Thermo Fisher Scientific). The cDNAs encoding for human *ZFX*, *UTY,* and *KDM6A* were purchased from Genescript and subcloned in the pTP6-3XT7 vector through EcoRI digestion. To obtain an inducible ZFX system, the pTP6-3XT7-ZFX was amplified using the primers described in **Supplemental Table S4** and subcloned into the inducible plasmid backbone PiggyBac (System Biosciences, PB210PA-1) through NheI and XhoI double digestion.

hESCs were transfected with either pTP6-3XFlag-KDM6A or PiggyBac-3XT7-ZFX plasmids (2.5 ug) in the presence of Lipofectamine 3000 (Thermo Fischer) to generate stable H1 KDM6A and ZFX overexpressing cells. To generate stable clones, hESCs were selected with 1 mg/ml Puromycin (Thermo Fischer) twenty-four hours after transfection. Individual clones were manually picked and transferred to a 96-well for further expansion. The inducible ZFX expression is obtained through treatment with Doxycycline at a concentration of 50 ng/mL for 48 hours.

To achieve the full deletion of *UTY,* we ordered a set of gRNAs from Genescript. The gRNAs were transfected by Neon electroporation together with Cas9 protein (PNABio, 5 mg/ml, CP02) into 47,XYY iPSCs. Cells were harvested 72 hours later and seeded at high dilution on Matrigel-coated plates. Individual clones were manually picked and transferred into 96-well for further Sanger sequencing validation and expansion. Each generated JS-iPSC UTY^-/-^ clone (N=4) used in this study was independently validated, and the editing of both *UTY* loci leading to premature translation termination was confirmed through Sanger sequencing (**Supplemental Fig S15**). We excluded any gRNA off-target on *KDM6A* by PCR amplification and Sanger sequencing. The tested gRNAs and oligos for the PCR-mediated amplification of the targeted genomic regions are listed in **Supplemental Table S4**.

**Immunofluorescence and image acquisitions**

iPSCs were plated on Matrigel-coated coverslips and fixed for 12 min with 3% paraformaldehyde for immunostaining 72 hours after seeding. Fixed cells were permeabilized with 0.25% Triton-X100 in PBS, incubated overnight with primary antibodies for pluripotency markers (**Supplemental Table S3**), washed, incubated with secondary antibodies, and mounted with ProLong Glass antifade Mounting solution with DAPI (Thermo Fisher Scientific, P36931). Immunostainings for pluripotency markers were acquired using an EVOS^TM^ FL Auto 2 Imaging System (Thermo Fisher Scientific) using a 1.30 NA/40X oil immersion objective (Olympus).

**RNA extraction and qPCR**

The total RNA extraction from iPSCs, fibroblasts, and NSCs was performed using the KingFisher Duo Prime benchtop sample extraction instrument (Thermo Fisher Scientific) using MagMAX mirVana Total RNA Isolation Kit (Applied Biosystems) following the manufacturer’s instructions. The cDNA was synthesized with the SuperScript VILO IV cDNA Synthesis Kit (Thermo Fisher Scientific). Gene expression was determined by real-time qPCR on a QuantStudio 3 Real-Time PCR System (Thermo Fisher Scientific) using TaqMan™ Fast Advanced Master Mix (Thermo Fisher Scientific) and TaqMan® Gene Expression Probes (**Supplemental Table S4**)**.** Individual gene expression was normalized on TATA Binding Protein (*TBP*) using the 2-ΔΔCt 2-(Delta Delta C(T)) as a relative quantification method.

**Whole Exome Sequencing and Allele-Specific Expression analysis**

According to manufacturer instructions, genomic DNA was extracted from hiPSC lines using the DNeasy Blood & Tissue Kits (Qiagen). The DNA quality was assessed using the TapeStation Agilent Genomic DNA ScreenTape Assay (Agilent) before DNA shipment. Beijing Novogene Bioinformatics Technology, Co., Ltd. (Beijing, China) performed the WES analysis. Target enrichment was performed to construct the exome library using the Agilent SureSelect Human All Exon V6 kit (Agilent Technologies, Inc., Santa Clara, CA, United States), according to the manufacturer’s protocol, and sequenced on Illumina Platform. An effective coverage of around 100× was obtained for all samples. Paired-end sequence reads for each individual were aligned to the GRCh38 reference human genome sequence using the sentieon bwa mem (BWA v0.7.17-r1188 aligner). Then, aligned reads were sorted, duplicates were marked using sentieon-Dedup, sorted with sentieon-sort, and base quality recalibrated using sention-ReadWriter (sention-genomics v202112.02), respectively. For the Variant Calling, a combination of GATK v4.2.6.1 SelectVariants, followed by sentieon TNfilter. We utilized the Sentineons’ TNhaplotyper2 algorithm with specific options to enhance accuracy.

The --call_germline_sites option directed the algorithm to call germline variants, while the --call_pon_sites option ensured using a panel of normal samples from the 1000 Genomes Project to classify common variants. Finally, bcftools 1.11+htslib-1.11 was used to keep the variants that met the following criteria: the FILTER field set as PASS or panel of normals and depth of coverage > 10. To ensure the accuracy of the called variants, we applied an additional filter to remove variants with no reads supporting either the reference or alternate alleles. We used ASERead Counter from GATK v4.2.2.0 for ASE analysis to obtain read counts covering each allele on SNPs. To detect SNPs differing from the reference genome, we set stringent parameters of base quality BQ ≥ 30, mapping quality MQ ≥ 10, and read depth > 6. We excluded intronic sites and non-uniquely mapped genes. Reference allele expression was calculated as reference allele counts divided by total counts. SNPs were classified as biallelic if reference allele expression was between 0.1 and 0.9 and monoallelic if outside this range. Genes were considered biallelic if at least one SNP in the gene was biallelic. Importantly, we detected a single biallelic expressed SNP on the X Chromosome in sample JS2 #A corresponding to the gene NUDT10. We took 50 bases upstream and downstream of the SNP and performed a blast against the RefSeq RNA database to investigate how this SNP could be found biallelic in a sample with only one X. We also visualized the bigWig files using the genomic viewer IGV(Robinson et al. 2011). We found a 100% match of the sequence against NUDT10 and a 99% with one mismatch against NUDT11. We concluded that the identified SNP, attributable to NUDT11, was mistakenly mapped to NUDT10, possibly due to a poorly mapping artifact resulting in a false SNP in one JS2 iPSC clone. The number of monoallelically expressed autosomal genes identified in our study is consistent with previous reports of random allelic expression (RAE) of autosomal genes in cell lines and primary tissues(Kravitz et al. 2023; Reinius and Sandberg 2015; Gendrel et al. 2016). Studies on human and mouse cell lines have documented random monoallelic expression in 1% to 15% of autosomal genes(Gendrel et al. 2016; Gimelbrant et al.). In our analysis, we observed an average of 4% of autosomal genes with monoallelic expression in fibroblasts, iPSCs, and NSCs (Supplemental Data S2).

**Y Chromosome transcriptome simulation**

To simulate RNA-seq reads from the Chromosome Y transcriptome, we first extracted the sequence of Chromosome Y and used the modified GTF (GRCh 38 Ensembl version 101) file to include transcripts only from Chromosome Y, also including the pseudoautosomal region (PAR) genes, which were sourced from Chromosome X due to masking of the Y Chromosome in the reference genome. We created a FASTA file using the gffread software v0.12.7(Pertea and Pertea 2020). Next, we simulated paired-end RNA-seq reads using the Modern ART (Artificial Read Transcription) software, an enhanced version of the ART software(Huang et al. 2011) in “transcriptome mode” to model realistic RNA-seq data. The simulation parameters were set as follows:

- **Mode:** --mode trans (for transcriptome)
- **Read type:** --lc pe (paired-end reads)
- **Read length:** --read_len 150 (150 bp per read)
- **Coverage:** --i-fcov 10 (10X unified coverage)
- **Fragment size:** --pe_frag_dist_mean 466 (mean fragment size of 466 bp)
- **Fragment size standard deviation:** --pe_frag_dist_std_dev 401 (standard deviation of 401 bp)

To further ensure the simulated reads reflected the sequencing platform characteristics, we generated Illumina-quality profiles for both read files (R1 and R2) using one random FASTQ sample from our data and the art_profiler_illumina script. This step was carried out to mimic the quality profile typically associated with Illumina Novaseq X sequencing.

The simulated reads were mapped to the GRCh38 human reference genome (Ensembl version 101) using the STAR aligner v2.7.10. Following the alignment, we used featureCounts (Subread suite v. 2.0.2) to count the reads mapped to genes. The featureCounts output provided the number of reads mapped to genes, which was subsequently analyzed to evaluate the accuracy and specificity of Y Chromosome read mapping. Our results indicate that the number of reads wrongly assigned to any other chromosome than Y is limited to 1 read count on Chromosome 6, corresponding to the pseudogene *CICP18*. As expected, the reads obtained from PAR genes were all univocally mapped to the X Chromosome.

**Multimapping assessment on JS-iPSC RNA-seq data**

To quantify the number of multi-mapping versus univocally assigned reads for NPY genes, we randomly selected one JS-iPSC transcriptome per patient (N=3). Next, we performed the following steps: first, reads were mapped to the GRCh38 human reference genome (Ensembl version 101) using the STAR aligner v2.7.10. Next, featureCounts (Subread suite v. 2.0.2) was used with the reference human GTF ensembl version 101 to count the reads mapped to genes; the read counting was performed in two modes: not counting multimapping reads and counting multimapping reads (-M mode) (**Supplemental Data S12**).

**Supplemental References**

Astro V, Adamo A. 2024. Generation of iPSC Cell Lines from Patients with Sex Chromosome Aneuploidies. In (ed. Springer), *Germ Cell Development*, pp. 185–200, Springer US, New York, NY <https://doi.org/10.1007/978-1-0716-3698-5_14>.

Gendrel A-V, Marion-Poll L, Katoh K, Heard E. 2016. Random monoallelic expression of genes on autosomes: Parallels with X-chromosome inactivation. *Semin Cell Dev Biol* **56**: 100–110.

Gimelbrant A, Hutchinson JN, Science BT, 2007. Widespread monoallelic expression on human autosomes. *science.sciencemag.org*. <https://science.sciencemag.org/content/318/5853/1136.short>.

Huang W, Li L, Myers JR, Marth GT. 2011. ART: a next-generation sequencing read simulator. *Bioinformatics* **28**: 593–594.

Kravitz SN, Ferris E, Love MI, Thomas A, Quinlan AR, Gregg C. 2023. Random allelic expression in the adult human body. *Cell Rep* **42**: 111945.

Pertea G, Pertea M. 2020. GFF Utilities: GffRead and GffCompare. *F1000Research* **9**: ISCB Comm J-304.

Pratt T, Sharp L, Nichols J, Price DJ, Mason JO. 2000. Embryonic stem cells and transgenic mice ubiquitously expressing a tau-tagged green fluorescent protein. *Developmental Biology* **228**: 19–28. <http://eutils.ncbi.nlm.nih.gov/entrez/eutils/elink.fcgi?dbfrom=pubmed&id=11087623&retmode=ref&cmd=prlinks>.

Reinius B, Sandberg R. 2015. Random monoallelic expression of autosomal genes: stochastic transcription and allele-level regulation. *Nat Rev Genet* **16**: 653–664.

Robinson JT, Thorvaldsdóttir H, Winckler W, Guttman M, Lander ES, Getz G, Mesirov JP. 2011. Integrative genomics viewer. *Nat Biotechnol* **29**: 24–26.
