# Supplementary material for: An iPSC-based model of 47,XYY Jacobs syndrome reveals a DNA methylation-independent transcriptional dysregulation shared with male X aneuploid cells
Source: Genome Res. 2025 Jul;35(7):1503–17. doi: 10.1101/gr.279716.124 (PMC12212075; doi:10.1101/gr.279716.124)
Supplement: Supplement 14 [file Supplemental_Table_S1_R3.docx]

| **Fibroblast cell line** | **Patient age (at sampling)** | **Ethnicity** | **Remarks** | **Repository** | **Name in the paper** | **Karyotype** |
| --- | --- | --- | --- | --- | --- | --- |
| GM01250 | 23 YR | Black/African American | Phenotypically normal | NIGMS Human Genetic | JS1 | 47,XYY |
| GM09326 | 2 Months | Irish/English | Phenotypically normal | NIGMS Human Genetic | JS2 | 47,XYY |
| GM11337 | 19 FW | N/A | Induced abortion | NIGMS Human Genetic | JS3 | 47,XYY |

**Supplemental Table S1. Fibroblast lines reprogrammed in this study.**

Description of 47,XYY Jacobs syndrome patient’ cohort
