# Supplementary material for: An iPSC-based model of 47,XYY Jacobs syndrome reveals a DNA methylation-independent transcriptional dysregulation shared with male X aneuploid cells
Source: Genome Res. 2025 Jul;35(7):1503–17. doi: 10.1101/gr.279716.124 (PMC12212075; doi:10.1101/gr.279716.124)
Supplement: Supplement 16 [file Supplemental_Table_S3_R3.docx]

**Antibodies used for Immunofluorescence and Western Blot.**

| **Antibody** | **Species** | **Dilution** | **Manufacturer** |
| --- | --- | --- | --- |
| NANOG | Rabbit | 1:200 | Abcam Cat# ab109250 |
| OCT4 | Mouse monoclonal | 1:100 | Thermo Fisher Scientific, Cat#MA1-104 |
| SOX2 | Mouse anti Rabbit | 1:500 | Cell Signalling Technology  Cat#46743S |
| SSEA4 | Mouse monoclonal | 1:100 | Thermo Fisher,  Cat#41-4000 |
| NESTIN | Rabbit monoclonal | 1:100 | Abcam  Cat#ab18102 |
| T7 | Rabbit | 1:2000 | Cell Signalling, Cat#13246 |
| GAPDH | Mouse monoclonal | 1:2000 | Abcam  Cat#ab8245 |
| FOXA2 | Rabbit | 1:100 | Abnova  Cat#H0003170-m10 |
| SOX17 | Mouse monoclonal | 1:100 | Abcam  Cat#ab84990 |
| SNAI1 | Rabbit | 1:100 | ProteinTech  Cat#13099-1-AP |
| N-Cadherin | Mouse monoclonal | 1:100 | Thermo Fisher Scientific  Cat#33-3900 |
| UTY (E3C2J) | Rabbit monoclonal | 1:1000 | Cell Signaling, Cat# 48779 |
| UTX | Rabbit | 1:500 | Sigma, Cat# SAB2702123 |
| Phalloidin Alexa Fluor® 647 | - | 1:200 | Thermo Fisher Scientific  Cat#A22287 |
| IgG H&L (Alexa Fluor® 647) | Goat anti mouse | 1:200 | Thermo Fisher Scientific  Cat# A21235 |
| IgG H&L (Alexa Fluor® 488) | Goat anti mouse | 1:200 | Thermo Fisher Scientific  Cat#A11029 |
| IgG H&L (Alexa Fluor® 568) | Goat-anti-rabbit | 1:200 | Thermo Fisher Scientific  Cat#A11036 |
| IgG H&L (Alexa Fluor®488) | Goat-anti-rabbit | 1:200 | Thermo Fisher Scientific  Cat#A11008 |
| IgG H&L (Alexa Fluor® 568) | Goat-anti-mouse | 1:200 | Thermo Fisher Scientific  Cat#A11004 |

**DNA-FISH probes used in this study.**

| Dual Labelled Satellite Probe Sets |
| --- |
| CytoCell, Cat#LPE 0XYc / LPE 0XYq |
