# Supplementary material for: An iPSC-based model of 47,XYY Jacobs syndrome reveals a DNA methylation-independent transcriptional dysregulation shared with male X aneuploid cells
Source: Genome Res. 2025 Jul;35(7):1503–17. doi: 10.1101/gr.279716.124 (PMC12212075; doi:10.1101/gr.279716.124)
Supplement: Supplement 17 [file Supplemental_Table_S4_R3.docx]

**Supplemental Table S4A: TaqMan Gene Expression probes**

| **TaqMan Assays** | **Manufacturer** | **Identifier** |
| --- | --- | --- |
| TaqMan qPCR assay: *TBP* | Thermo Fisher Scientific | Cat#Hs00427620_m1 |
| TaqMan qPCR assay: *OCT4* | Thermo Fisher Scientific | Cat#Hs04260367_gH |
| TaqMan qPCR assay: *NANOG* | Thermo Fisher Scientific | Cat#Hs02387400_g1 |
| TaqMan qPCR assay: *NES* | Thermo Fisher Scientific | Cat#Hs04187831_g1 |
| TaqMan qPCR assay: *TUBB3* | Thermo Fisher Scientific | Cat#Hs00801390_s1 |
| TaqMan qPCR assay: *KDM5D* | Thermo Fisher Scientific | Cat#Hs00190491_m1 |
| TaqMan qPCR assay: *UTY* | Thermo Fisher Scientific | Cat#Hs01076483_m1 |
| TaqMan qPCR assay: *DDX3Y* | Thermo Fisher Scientific | Cat#Hs00606179_m1 |
| TaqMan qPCR assay: *NLGN4Y* | Thermo Fisher Scientific | Cat#Hs0103434378_s1 |
| TaqMan qPCR assay: *NLGN4X* | Thermo Fisher Scientific | Cat#Hs01934144_s1 |
| TaqMan qPCR assay: *DDX3X* | Thermo Fisher Scientific | Cat#Hs00606179_m1 |
| TaqMan qPCR assay: *UTX* | Thermo Fisher Scientific | Cat#Hs00958902_m1 |
| TaqMan qPCR assay: *ZFX* | Thermo Fisher Scientific | Cat#Hs01017881_m1 |
| TaqMan qPCR assay: *SOX2* | Thermo Fisher Scientific | Cat#Hs01053049_s1 |

**Supplemental Table S4B: Oligos used for real-time PCR**

| **Oligo Names** | **Manufacturer** | **Exon location** | **Identifier** |
| --- | --- | --- | --- |
| UTX/KDM6A | Sigma-Aldrich | 19-22 | Cat#8821625448-000010/20 |

**Supplemental Table S4C: Oligos used ZFX cDNA amplification prior subcloning**

| **Oligo Names** | **Manufacturer** | **Sequence (5’-3’)** |
| --- | --- | --- |
| ZFX-NheI-FWD | Sigma Custom Oligos | AGCTAGCCATCATTTTGGCAAAG |
| ZFX-XhoI-R | Sigma Custom Oligos | ATATCTCGAGAGAATTCTTAGGGCAG |

**Supplemental Table S4D: siRNAs used for silencing**

| **siRNA Names** | **Manufacturer** | **Sequence** |
| --- | --- | --- |
| hs.Ri.UTY.13.2 | IDT (Integrated DNA technologies) | Not available |
| hs.Ri.ZFY.13.1 | IDT (Integrated DNA technologies) | Not available |
| hs.Ri.DDX3Y.13.3 | IDT (Integrated DNA technologies) | Not available |
| hs.Ri.NLGN4Y.13.2 human | IDT (Integrated DNA technologies) | Not available |
| Human Scrambled Negative Control DsiRNA | IDT (Integrated DNA technologies) | Catalog # 51-01-19-09 |

**Supplemental Table S4E: oligos used for Cleavage genomic detection in the CRISPR-Cas9 experiment**

| **Oligo Names** | **Manufacturer** | **Target** | **Sequence (5’-3’)** |
| --- | --- | --- | --- |
| UTY-2 Pr1 LeftPrimer | GenScript | *UTY* | AGCCTGACAGTCGAGGAAAG |
| UTY-2 Pr1 RightPrimer | GenScript | *UTY* | ATACTGGCTGGGCGGTAAGG |
| Primer 1F GCD exon 2 | Sigma Custom Oligos | *UTX* | ACCTCGGTTTGGCGCTCTTC |
| Primer 1R GCD exon 2 | Sigma Custom Oligos | *UTX* | ATCTGAACACACTCACGATC |

**Supplemental Table S4F: gRNAs tested for CRISPR-Cas9 mediated UTY KO experiments**

| **gRNA** | **Manufacturer** | **Sequence 5'-3'** | **PAM** | **Cat. #** | ***hUTY* Genomic Location** |
| --- | --- | --- | --- | --- | --- |
| gRNA UTY-1 | GenScript | GTCTGTTAGCCTGACAGTCG | AGG | EasyEdit sgRNA UTY-1 | 13479542..13479564 |
| gRNA UTY-2 | GenScript | GCCTCACGAACCCGAAGAGA | CGG | EasyEdit sgRNA UTY-2 | 13479295..13479317 |
| gRNA UTY-3 | GenScript | ATCACCGAAGGCAACAGCGG | CGG | EasyEdit sgRNA UTY-3 | 13479615..13479637 |
