# Supplementary material for: An iPSC-based model of 47,XYY Jacobs syndrome reveals a DNA methylation-independent transcriptional dysregulation shared with male X aneuploid cells
Source: Genome Res. 2025 Jul;35(7):1503–17. doi: 10.1101/gr.279716.124 (PMC12212075; doi:10.1101/gr.279716.124)
Supplement: Supplement 18 [file Supplemental_Figures.pdf]

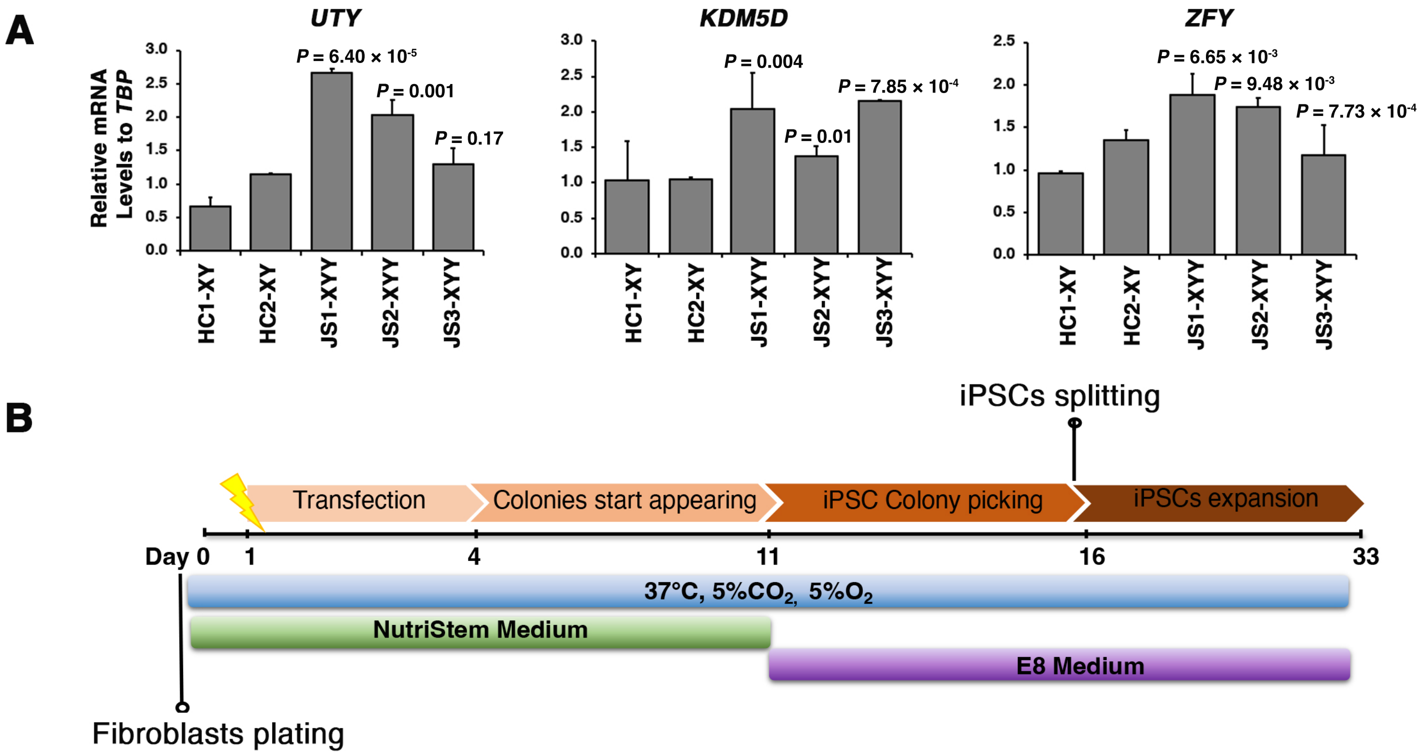

Supplemental_Fig_S1

Supplemental Fig S1: 47,XYY Fibroblasts reprogramming into iPSCs. A) mRNA expression of the *UTY*, *ZFY,* and *KDM5D* Y-linked genes by Taqman assay in 47,XYY and 46,XY fibroblasts. Values are normalized on the housekeeping gene TATA box-binding protein (*TBP*). Bars are the average ± std of three independent experiments for each iPSC clone. Values from two 46,XY control's fibroblasts were merged (n=6) to perform a Student's *t*-test, one-tailed distribution, two-sample equal variance. HC, healthy controls 46,XY. **B)** Schematic of the mRNA non-integrative reprogramming method used in the study.

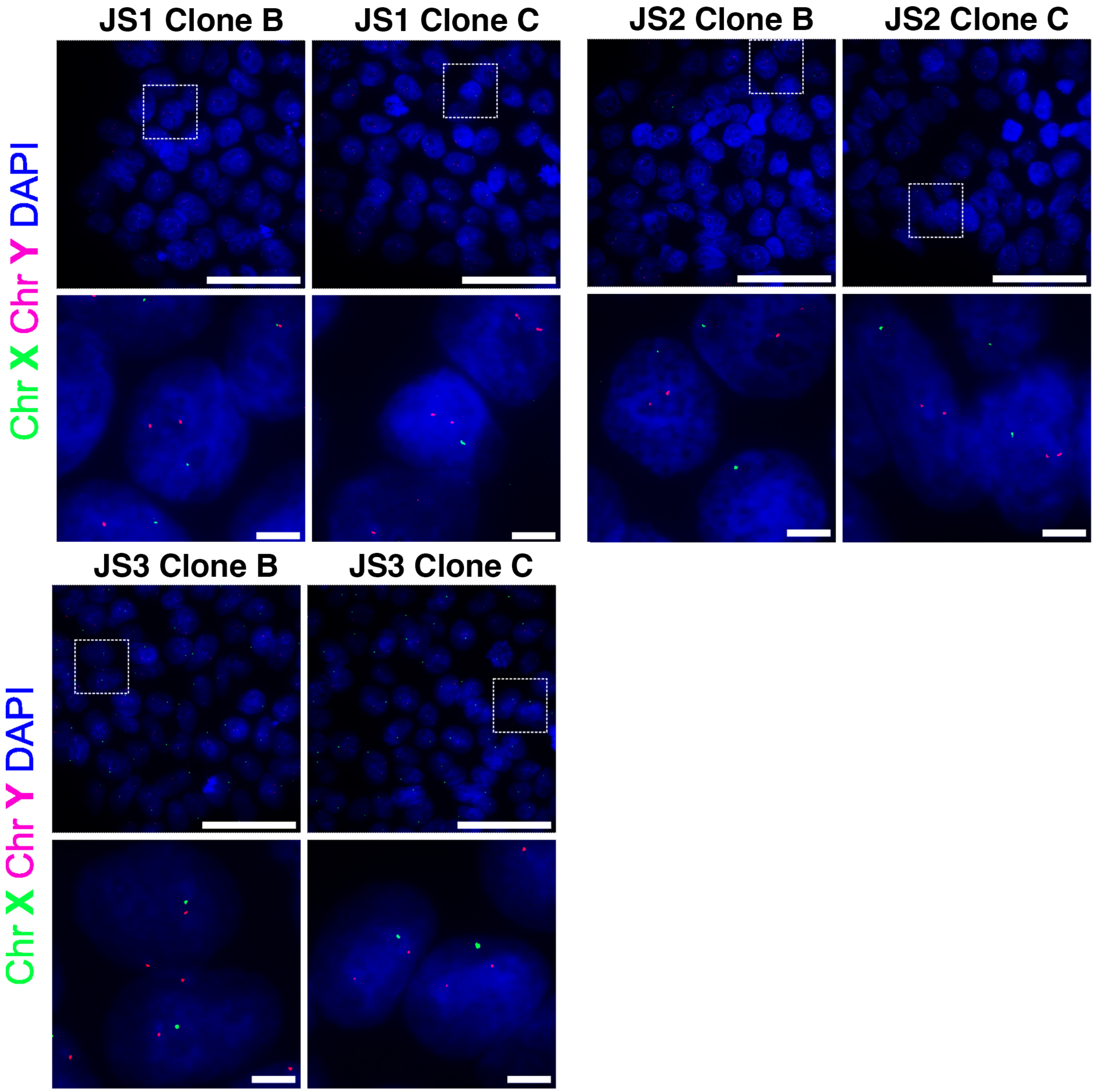

Supplemental_Fig_S2

Supplemental Fig S2: Validation by DNA-FISH of Y Chromosome copy gain in iPSCs. Top: Representative DNA-FISH images showing the X Chromosome (green) and Y Chromosome (red) in JS-iPSCs. DNA is stained with DAPI (blue). Scale bar = 50 µm. Bottom: Magnified view of the dashed square in the top image. Scale bar = 5 µm.

A

Clone name	Chromosome	Type	Cytoband Start	CN State	Size (kbp)
JS1 Clone A	Y	Gain	p11.2	2.00	18,642
	Y-X PAR1	Gain	p22.33	3.00	2,111
JS1 Clone B	Y	Gain	p11.2	2.00	19,068
	Y-X PAR1	Gain	p22.33	3.00	2,035
	11	Gain	q13.5	3.00	1,218
JS1 Clone C	Y	Gain	p11.2	2.00	18,604
	Y-X PAR1	Gain	p22.33	3.00	1,504
JS2 Clone A	Y	Gain	p11.2	2.00	23,624
	Y-X PAR1	Gain	p22.33	3.00	2,111
JS2 Clone B	Y	Gain	p11.2	2.00	23,646
	Y-X PAR1	Gain	p22.33	3.00	2,525
	6	Gain	q25.1	3.00	1,998
JS2 Clone C	Y	Gain	p11.2	2.00	23,615
	Y-X PAR1	Gain	p22.33	3.00	1,513
JS3 Clone A	Y	Gain	p11.2	2.00	21,481
	Y-X PAR1	Gain	p22.33	3.00	1,643
JS3 Clone B	Y	Gain	p11.2	2.00	19,068
	Y-X PAR1	Gain	p22.33	3.00	1,818
JS3 Clone C	Y	Gain	p11.2	2.00	21,481
	Y-X PAR1	Gain	p22.33	3.00	1,643

B

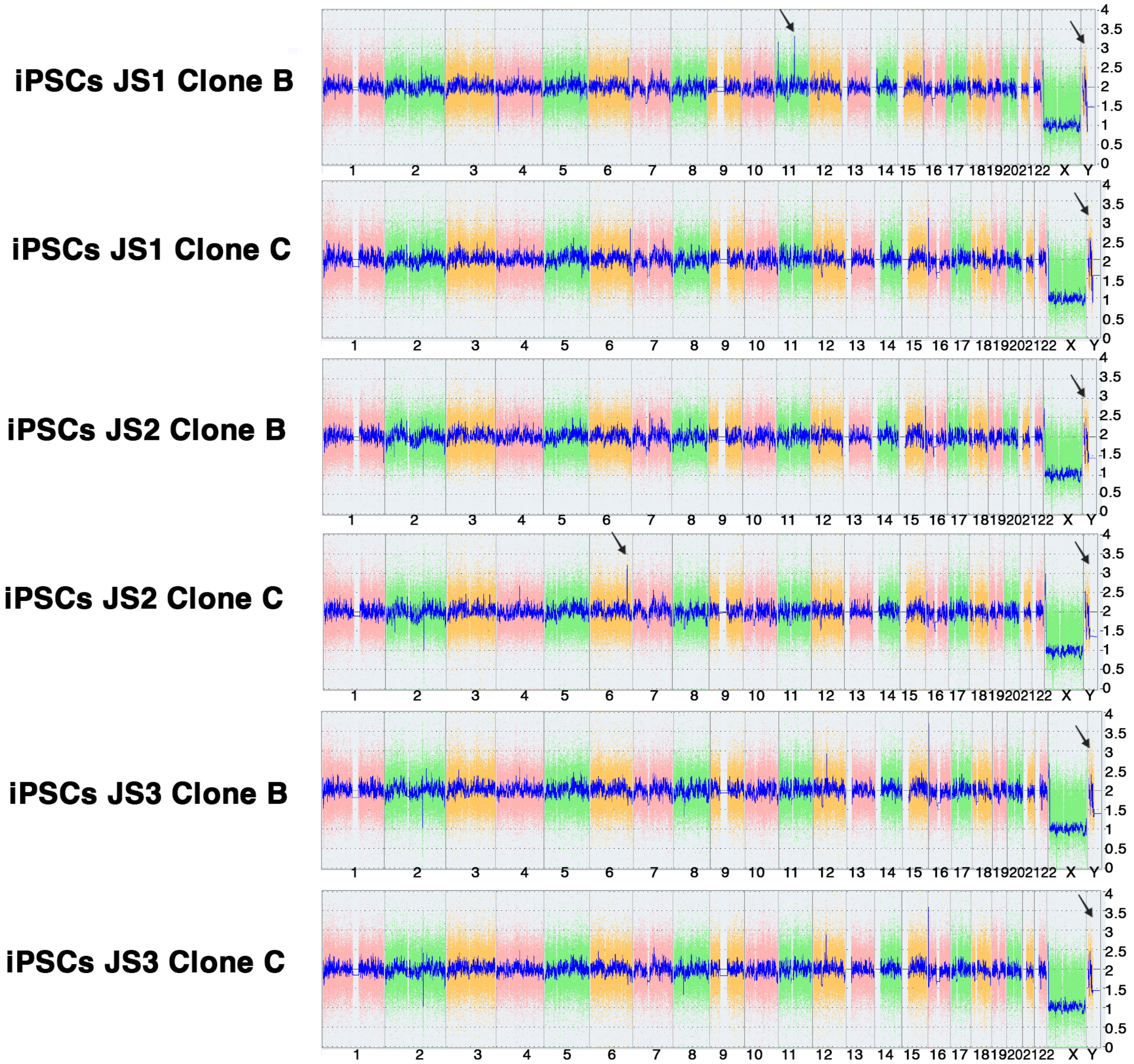

Supplemental_Fig_S3

Supplemental Fig S3: Karyostat arrays on 47-XXY iPSCs. A) Summary of the validated iPSCs. The probe binding to Xp22.33 also recognizes the Yp11.2 region and corresponds to the PAR1 territory on X and Y Chromosome, respectively; therefore, the showed X Chromosome's duplication for this region is an artifact of the probe binding to the PAR1 region of both sex chromosomes. The results show two copy number state (CN = 2) for the whole Y Chromosome except for the Yp11.2 probe (CN = 1) automatically assigned to the X Chromosome (Xp22.33, CN = 3). **B)** KaryoStat+ whole genome view**.** The pink, green, and yellow colors indicate the raw signal for each chromosome probe, while the blue represents the normalized probe signal, which is used to identify copy numbers and aberrations. Black arrows indicate chromosome gains.

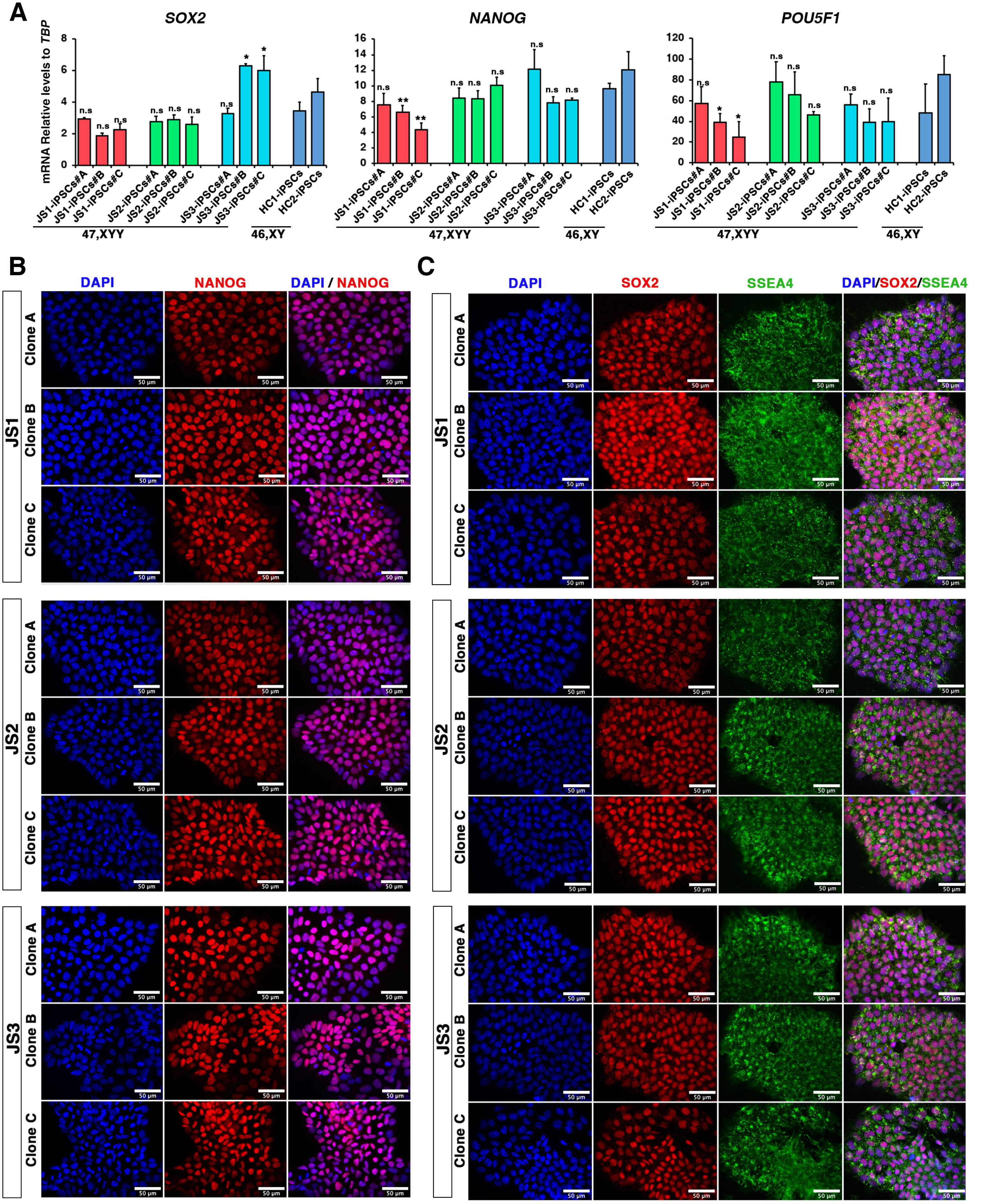

Supplemental_Fig_S4

Supplemental Fig S4: Pluripotency characterization by immunostaining on 47,XYY iPSCs generated in this study. A) Transcript levels of the pluripotency markers *SOX2*, *NANOG,* and *POUF5F1* in the iPSCs generated in the study analyzed by TaqMan Assay. Bars are the average ± std of three independent experiments. Values from two 46,XY control's iPSCs were merged (n=6) to perform a Student's *t*-test, one-tailed distribution, and two-sample equal variance. $^{*}P < 0.05$, $^{**}P < 0.01$, n.s. = not significant. **B-C)** Staining for the pluripotency markers NANOG (red), SOX2 (red), and SSEA4 (green). See **Supplemental Table S11** for antibody information. DAPI (blue) is used as a counterstain to detect nuclei. Images have been acquired using an EVOS^{TM} FL Auto 2 Imaging System (Thermo Fisher Scientific) equipped with a 1.30NA/40X oil immersion objective (Olympus). Scale bars, 50 μm.

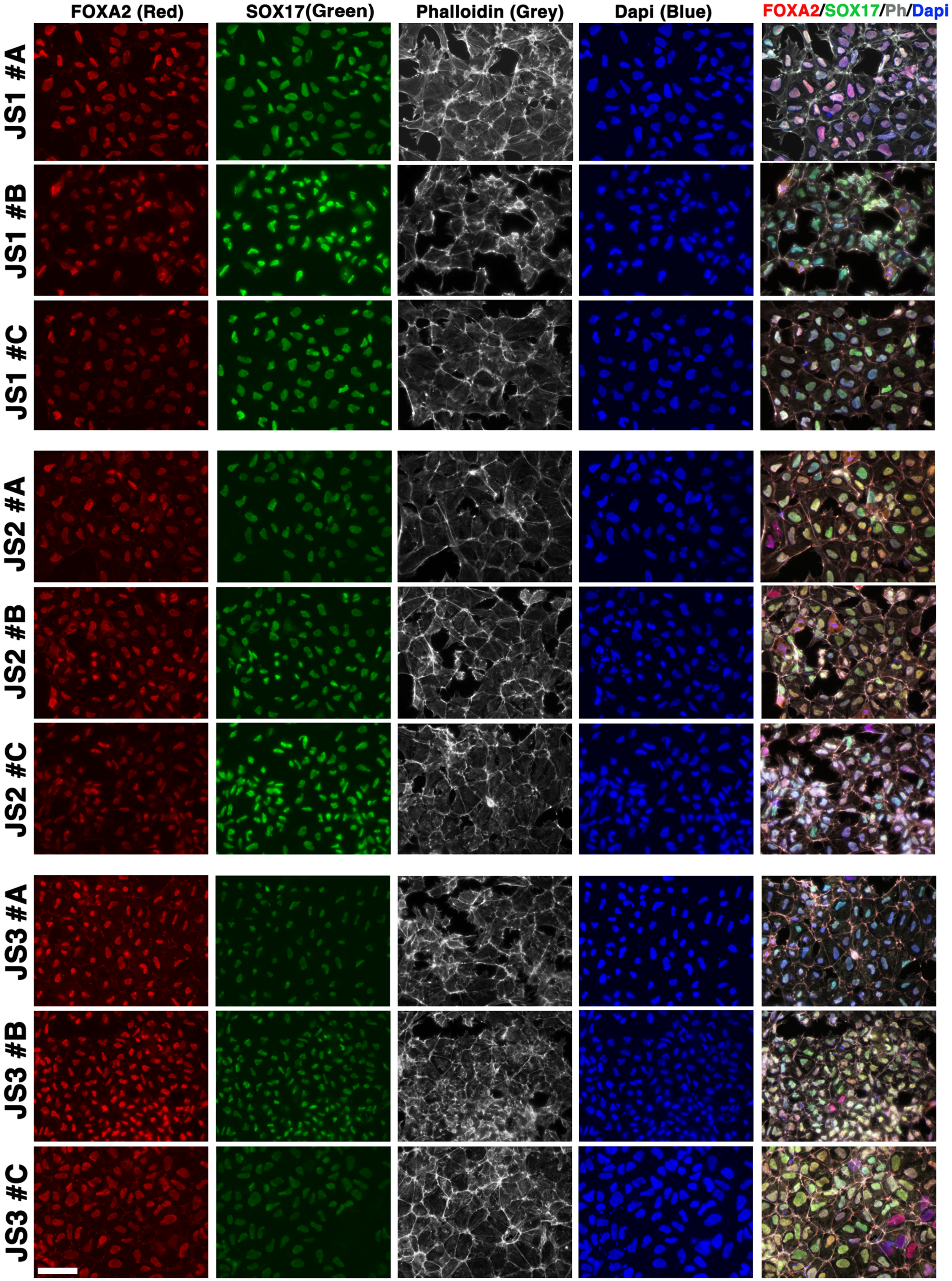

Supplemental_Fig_S5

Supplemental Fig S5: Differentiation into endoderm derivatives. Immunostaining of 47,XYY iPSCs differentiated into definitive endoderm to assess their differentiation potential. Cells have been labeled for FOXA2 (red), SOX17 (green), and Phalloidin (grey). Nuclei are counterstained with DAPI (Blue). Scale bar, 50 μm.

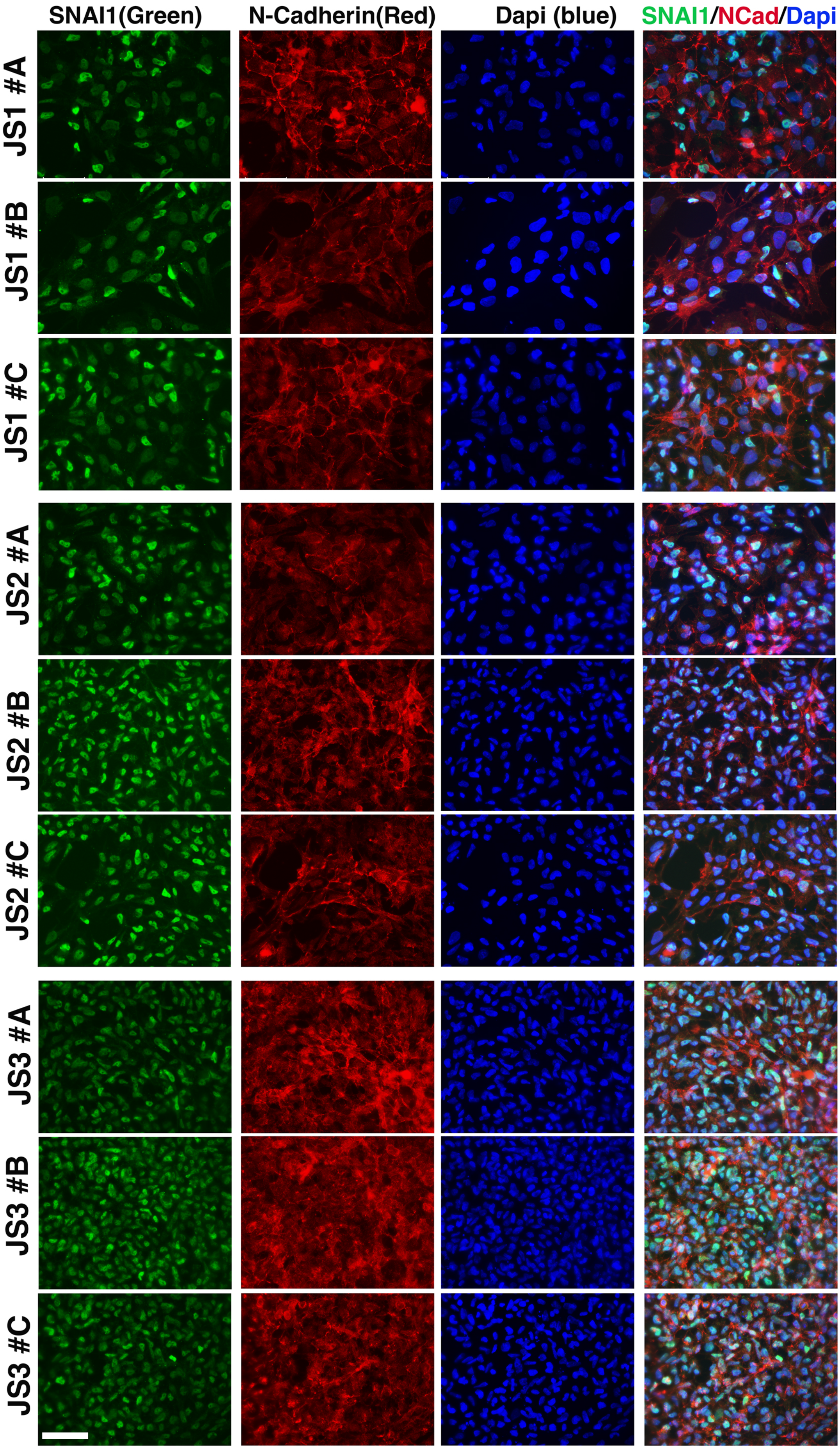

Supplemental_Fig_S6

Supplemental Fig S6: Differentiation into Mesoderm lineage. Immunostaining of 47,XYY iPSCs differentiated into cardiac mesoderm to assess their differentiation potential. Cells have been labeled for the mesodermal markers SNAI1 (green), N-Cadherin (red), and DAPI (Blue). Scale bar, 50 μm.

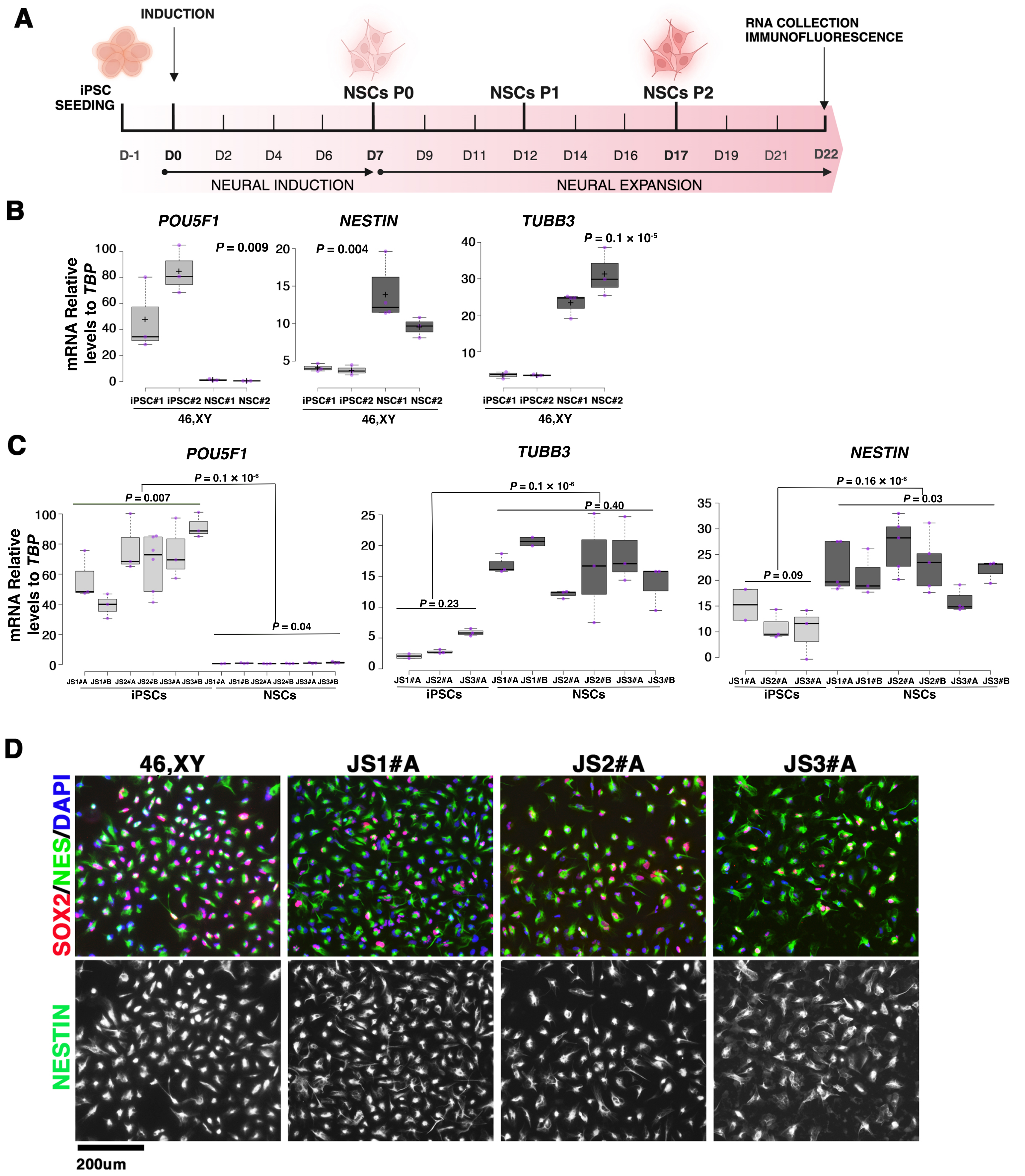

Supplemental_Fig_S7

Supplemental Fig S7: Derivation of neural stem cells from 47,XYY and 46,XY iPSCs. **A)** Timeline of iPSCs differentiation into NSCs. At approximately 20-22 days of differentiation, NSCs reached passage 2 (P2) and were collected for RNA profiling or Immunofluorescence. **B-C)** Taqman assay showing the mRNA expression of the pluripotency marker *POU5F1*, and NSC markers *NESTIN* (*NES*) and *TUBULIN3* (*TUBB3*) in 46,XY (B) or 47,XYY cells (C). Values are normalized to the internal control TATA-binding protein (*TBP*). Each Purple dot represents an independent qPCR sample. One-way ANOVA and post-hoc Tukey HSD analyses were used to test the significance between groups. *P* values are shown. **D)** 47,XYY NSCs stained for the indicated markers. Images have been acquired using an EVOS™ FL Auto 2 Imaging System equipped with a 0.3NA/10X oil immersion objective (Olympus). Scale bars, 200 μm. See **Supplemental Table S11** for antibody information.

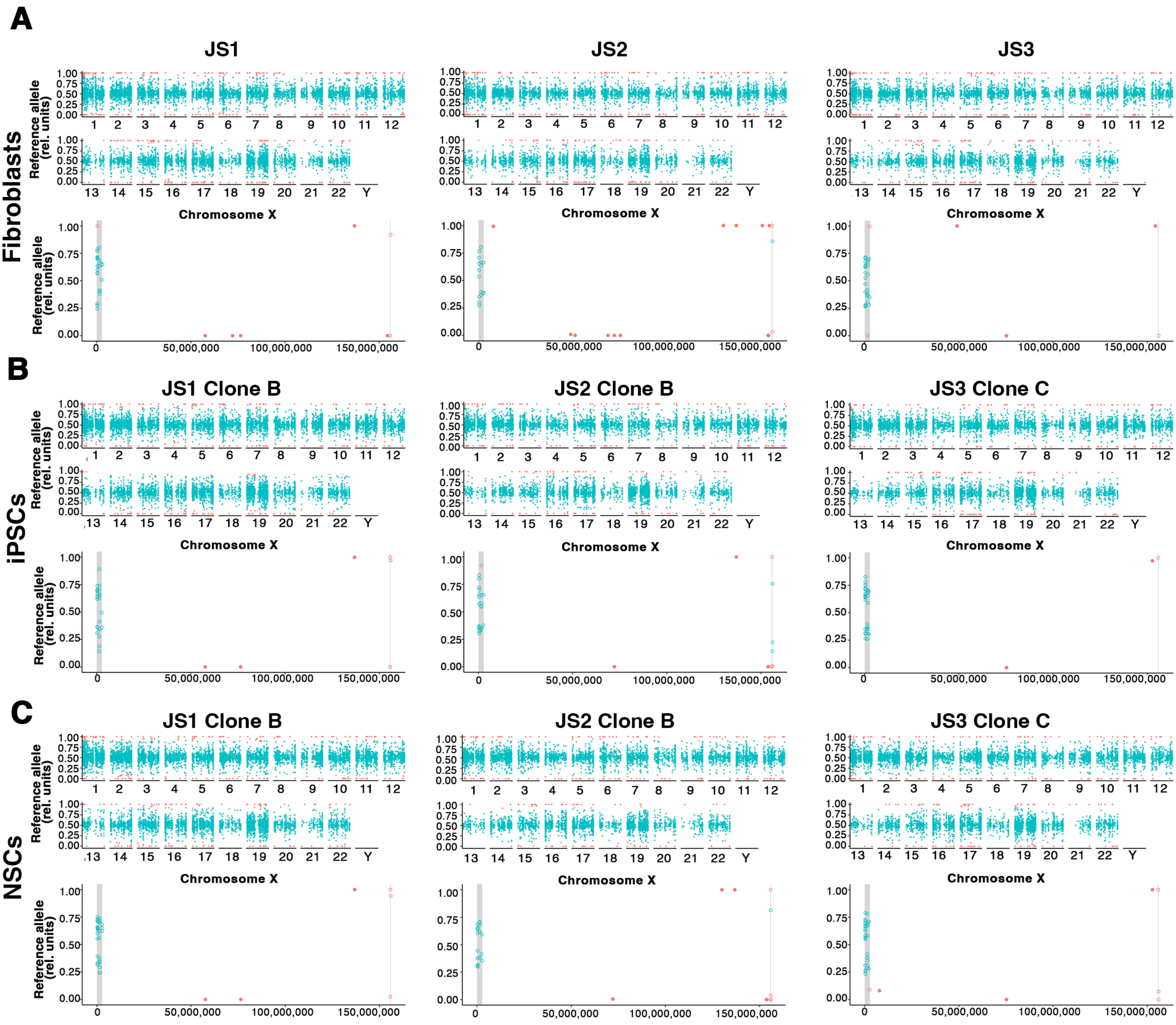

Supplemental_Fig_S8

Supplemental Fig S8: Allele-specific expression (ASE) analysis shows the biallelic expression of SNPs at the PAR region in 47,XYY cells. Scatter plot profiles of coupled WES analysis and allele-specific RNA-seq analysis performed on autosomal (upper panel) and X Chromosomes (lower panel) showing the mono- (orange dots) or biallelic (light blue dots) gene expression status in 47,XYY iPSCs. Gray rectangles indicate PAR1 and PAR2 regions, respectively. Solid dots indicate non-PAR genes; open dots show PAR genes.

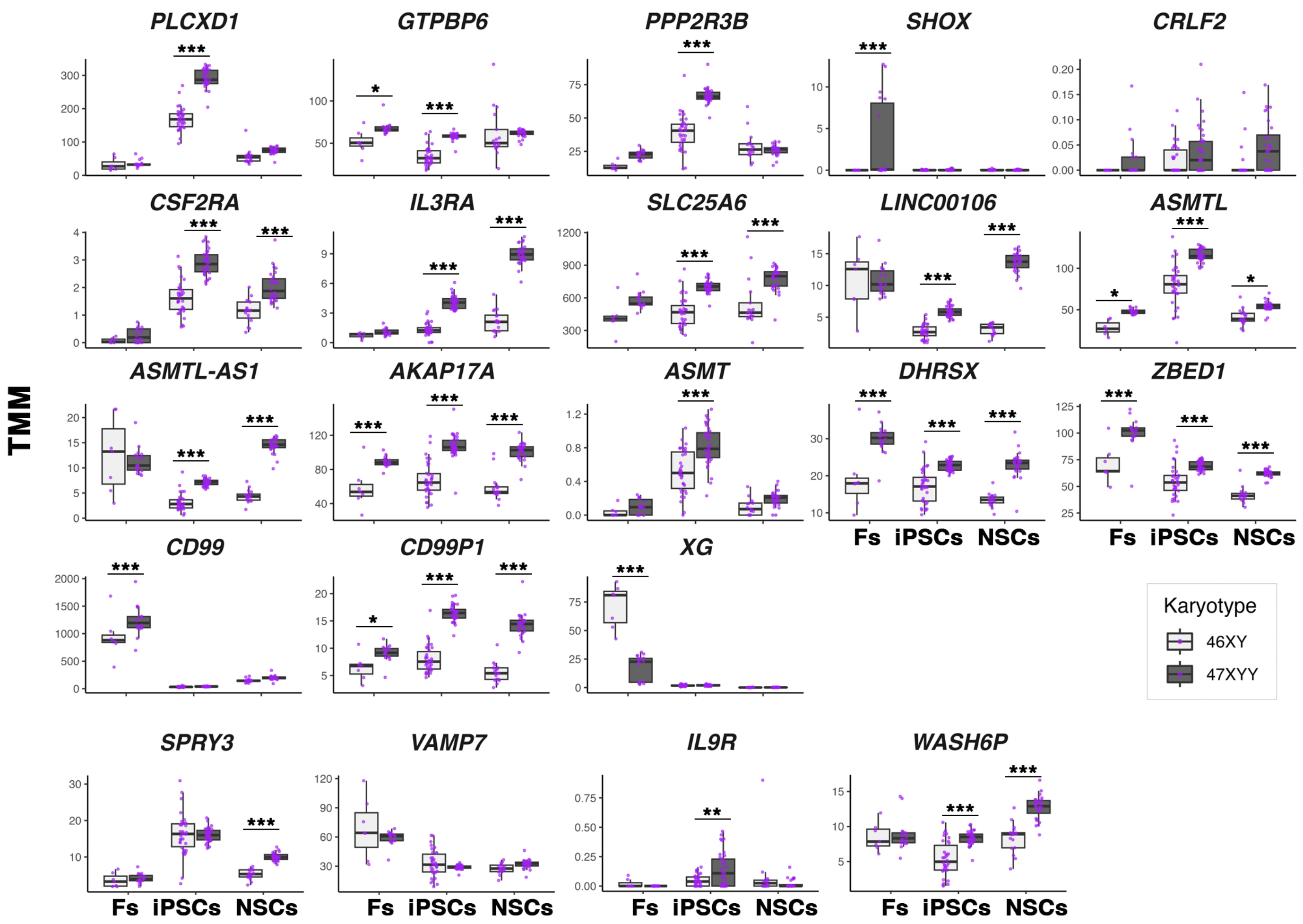

Supplemental_Fig_S9

Supplemental Fig S9: Signature of cell-type specific PAR expression. Box Plot of normalized TMM expression of PAR genes in control 46,XY and 47,XYY Fibroblasts, iPSCs, and NSCs. Each purple dot represents an independent RNA sample. Two-way ANOVA and post-hoc Tukey HSD analyses were used to test the significance of the groups and karyotypes. **P* value < 0.05; ***P* value < 0.01; ****P* value < 0.001.

A B C
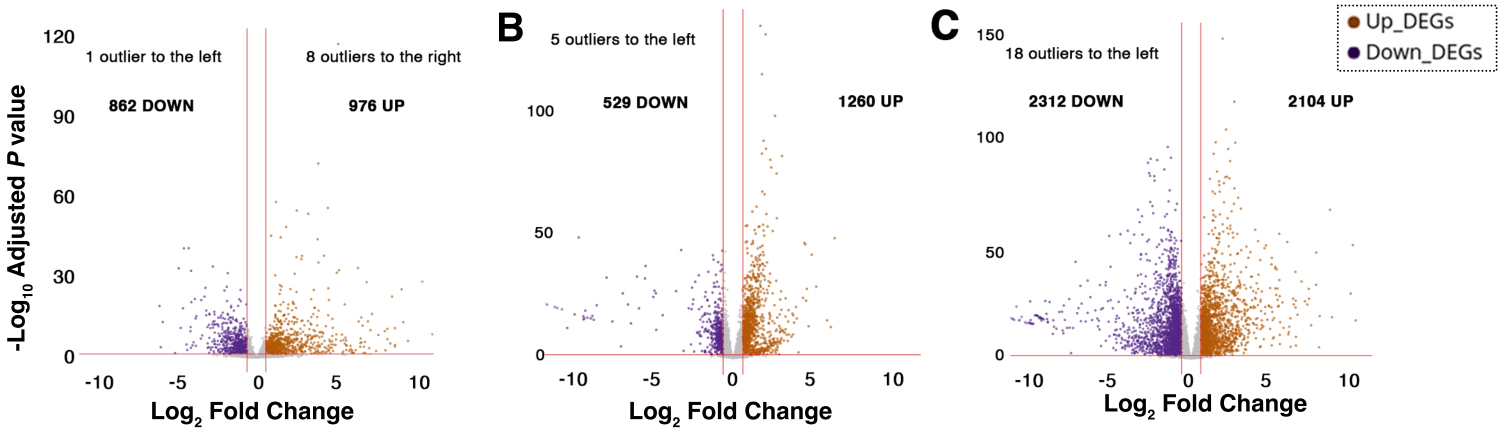

D

	47,XYY Vs. 46,XY (N.DEGs)		
	Fibroblasts	iPSCs	NSCs
Autosomes	1769	1696	4229
Sex Chromosomes	69	89	181
X Chromosome (non-PAR)	50	62	162
Y Chromosome (non-PAR)	14	15	12
X and Y PAR genes	5	12	7

Supplemental_Fig_S10

Supplemental Fig S10: Genome-wide effects of Y Chromosome aneuploidy. A-C) Volcano plots effect size and significance of DEGs detected in 47,XYY versus 46,XY (A) Fibroblasts, (B) iPSCs, and (C) NSCs. The numbers of up and down-regulated DEGs are shown for each contrast. **D)** Summary of the autosomal, PAR, and non-PAR X and Y DEGs detected in the three cell types. FDR<0.05 and $Log_2FC > |0.58|$.

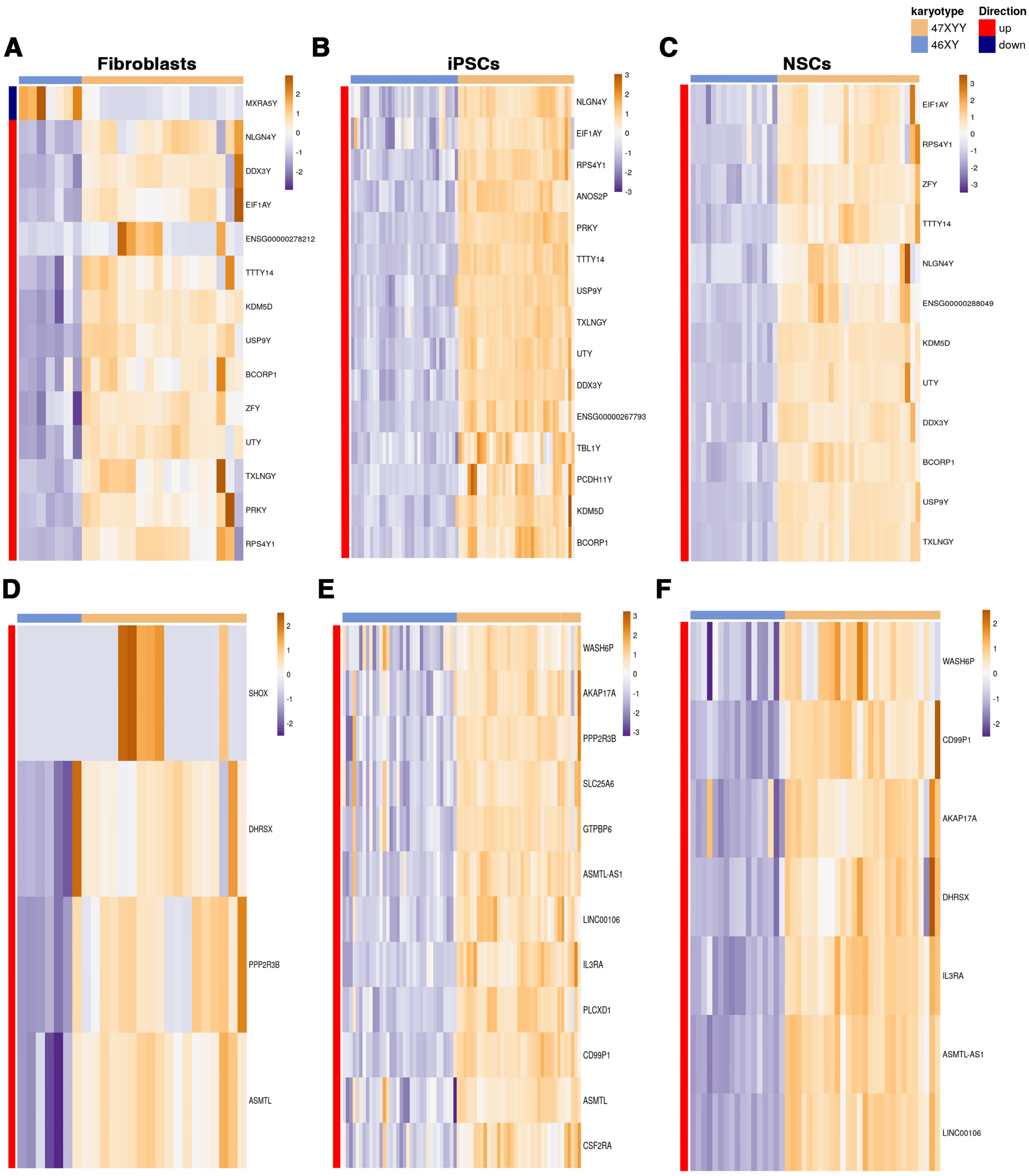

Supplemental_Fig_S11

Supplemental Fig S11: Overdosage of PAR and NPY genes in Y Chromosome aneuploid cells. A-C) Heatmap showing the TMM expression of upregulated NPY DEGs in 47,XYY vs. 46,XY contrast in (A) Fibroblasts, (B) iPSCs, and (C) NSCs. **D-F)** Heatmap showing the TMM expression of upregulated PAR DEGs in (D) Fibroblasts, (E) iPSCs, and (F) NSCs. TMM, Trimmed Mean of M-values.

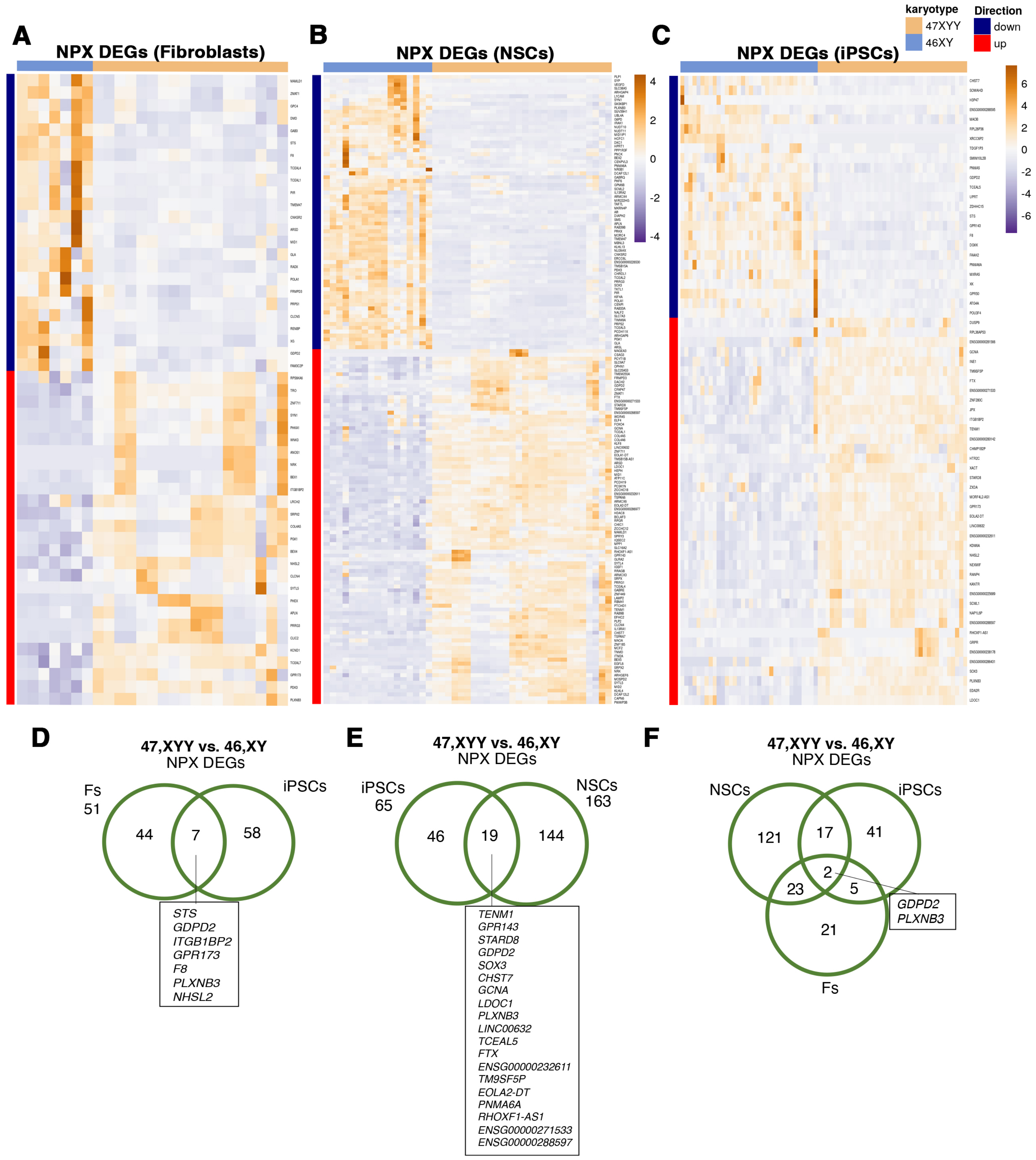

Supplemental_Fig_S12

Supplemental Fig S12: Y-linked gene overexpression modulates X Chromosome expression. A-C) Heatmap showing the TMM expression of up- and downregulated NPX DEGs in 47,XYY (A) Fibroblasts, (B) NSCs, and (C) iPSCs. **D-F)** Venn diagrams of shared NPX DEGs in (D) fibroblasts and iPSCs, (E) iPSCs and NSCs, or (F) the three cell types. The number of NPX genes is shown in each diagram.

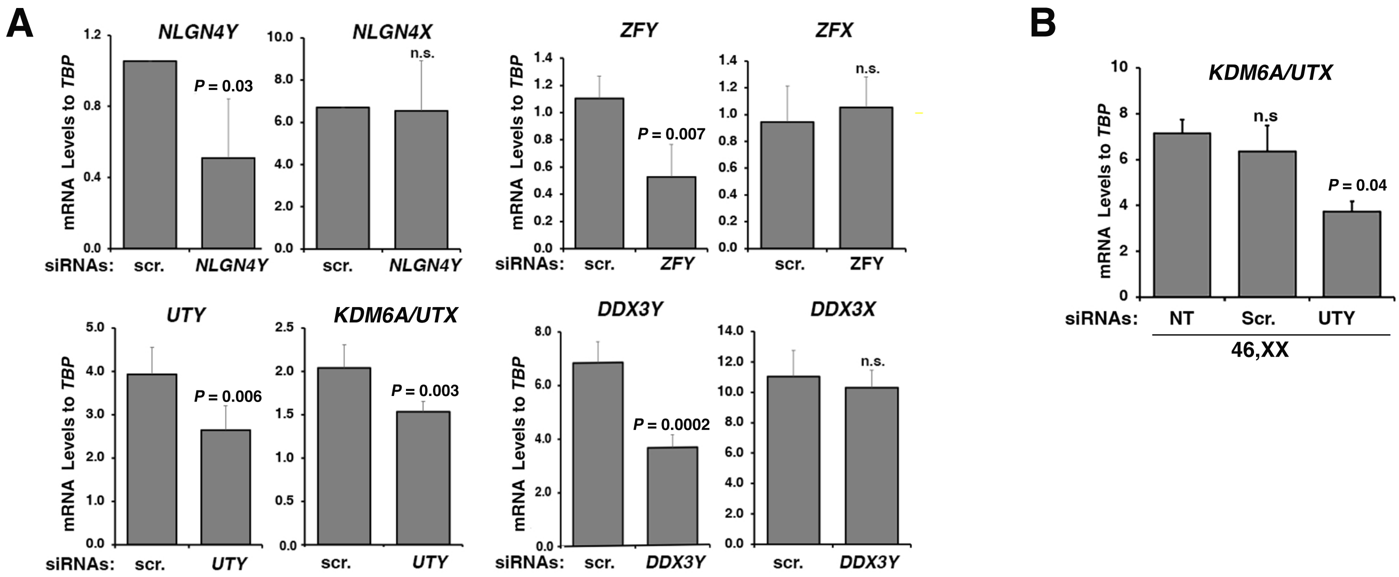

Supplemental _Fig_S13

Supplemental Fig S13: siRNA perturbation experiments on selected NPY genes. A) 47,XYY iPSCs have been transfected with siRNA against *ZFY*, *DDX3Y*, *NLGN4Y*, and *UTY*. Taqman assays are used to analyze the expression of the indicated mRNAs in control cells transfected with either siRNA scramble (scr.) or siRNAs specific to the indicated targets. Bars represent the average ± std of four independent experiments on iPSC clones derived from the three patients. **B)** The siRNA against *UTY* tested in 46,XX iPSCs has shown unspecific targeting of *KDM6A*. Bars represent the average ± std of two independent experiments. Student's *t*-test, one-tailed distribution, two-sample unequal variance. *P* values are indicated.

A

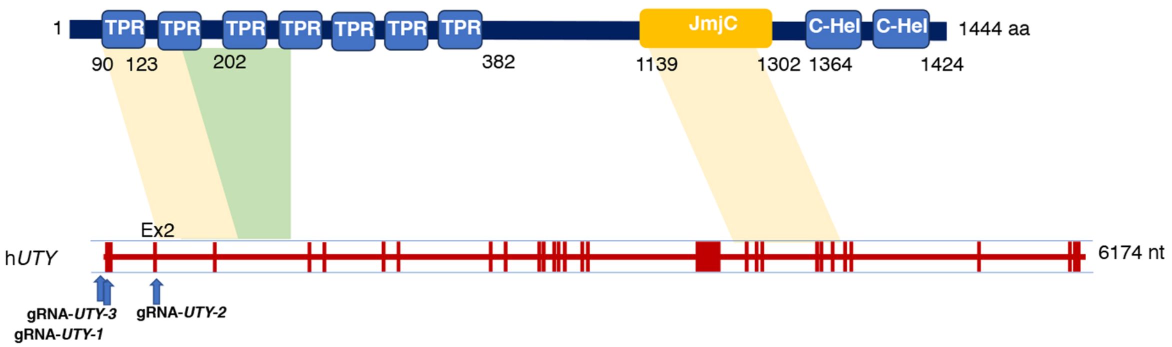

B

gRNA Name	Gene Symbol	Sequence 5'-3'	PAM	Strand	*hUTY* Genomic Location	On Target Score	Off Target Score	Overall Score	*In vitro* Tested Indel %
gRNA UTY-1	*UTY*	GTCTGTTAGCCTGACAGTCG	AGG	-	13479542..13479564	0.53	0.09	66.00	45%
gRNA UTY-2	*UTY*	GCCTCACGAACCCGAAGAGA	CGG	+	13479295..13479317	0.94	0.19	66.00	57%
gRNA UTY-3	*UTY*	ATCACCGAAGGCAACAGCGG	CGG	+	13479615..13479637	1.29	0.53	52.00	58%

C

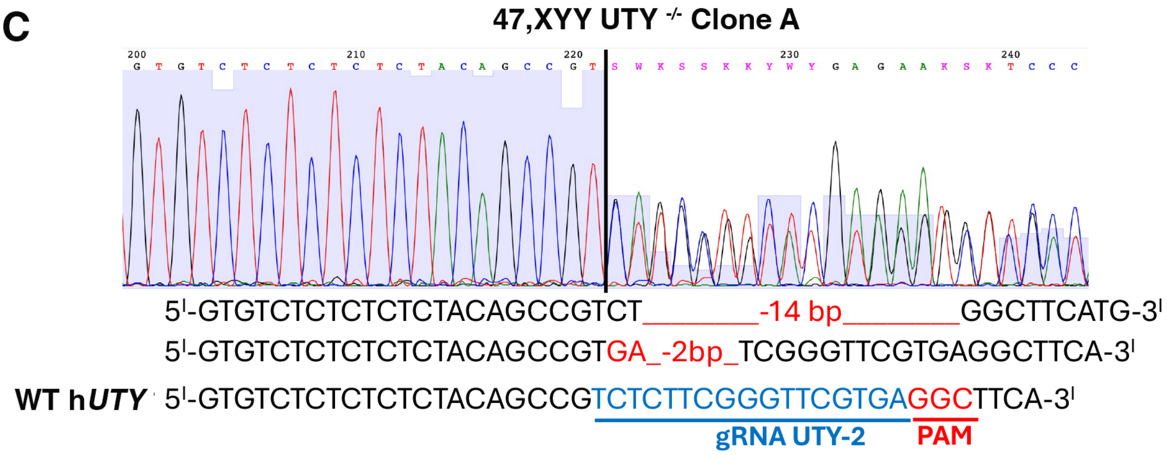

D

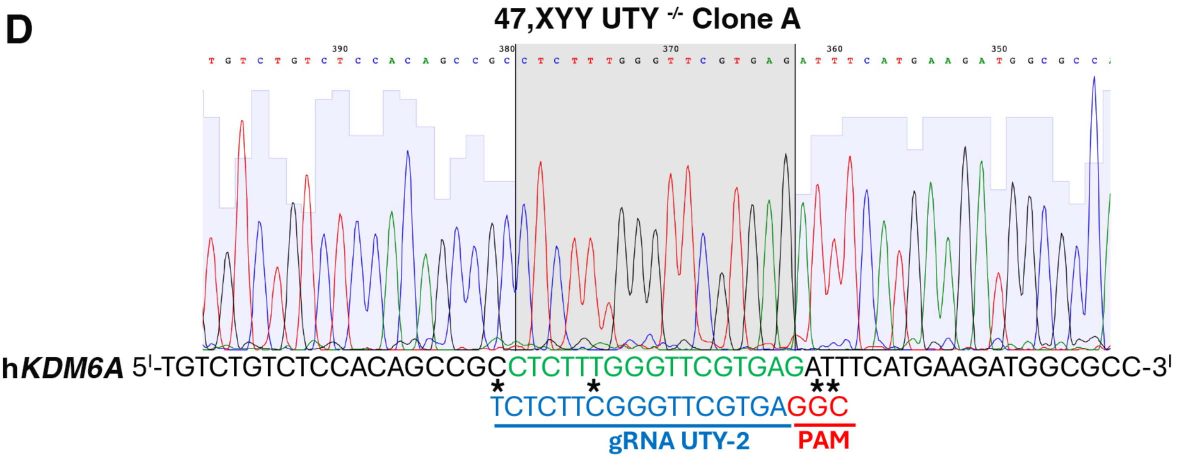

Supplemental_Fig_S14

Supplemental Fig S14: Generation of 47,XYY UTY Knock-out iPSCs. A) Schematic of UTY protein (top) and gene (bottom). Tested guide RNAs (gRNAs) targeting *UTY* exon 1 and exon 2. TPR, tetratricopeptide repeat; JmjC, jumonji C domain; C-Hel, C-terminal helical domain **B)** gRNA targeting the human *UTY* sequence (NC_000024.10:c13480670-13233895 hUTY). gRNA sequence, on target, off targets and Indel score are shown. **C)** Representative electropherogram of the *UTY* edited sequence in one 47,XYY UTY $^{-/-}$ iPSCs using gRNA UTY-2. **D**) The gRNA UTY-2 is not editing the *KDM6A* sequence. Electropherogram of *KDM6A* in a representative 47,XYY UTY $^{-/-}$ clone. Asterisks indicate mismatches between the gRNA targeting *UTY* and *KDM6A* exon 2.

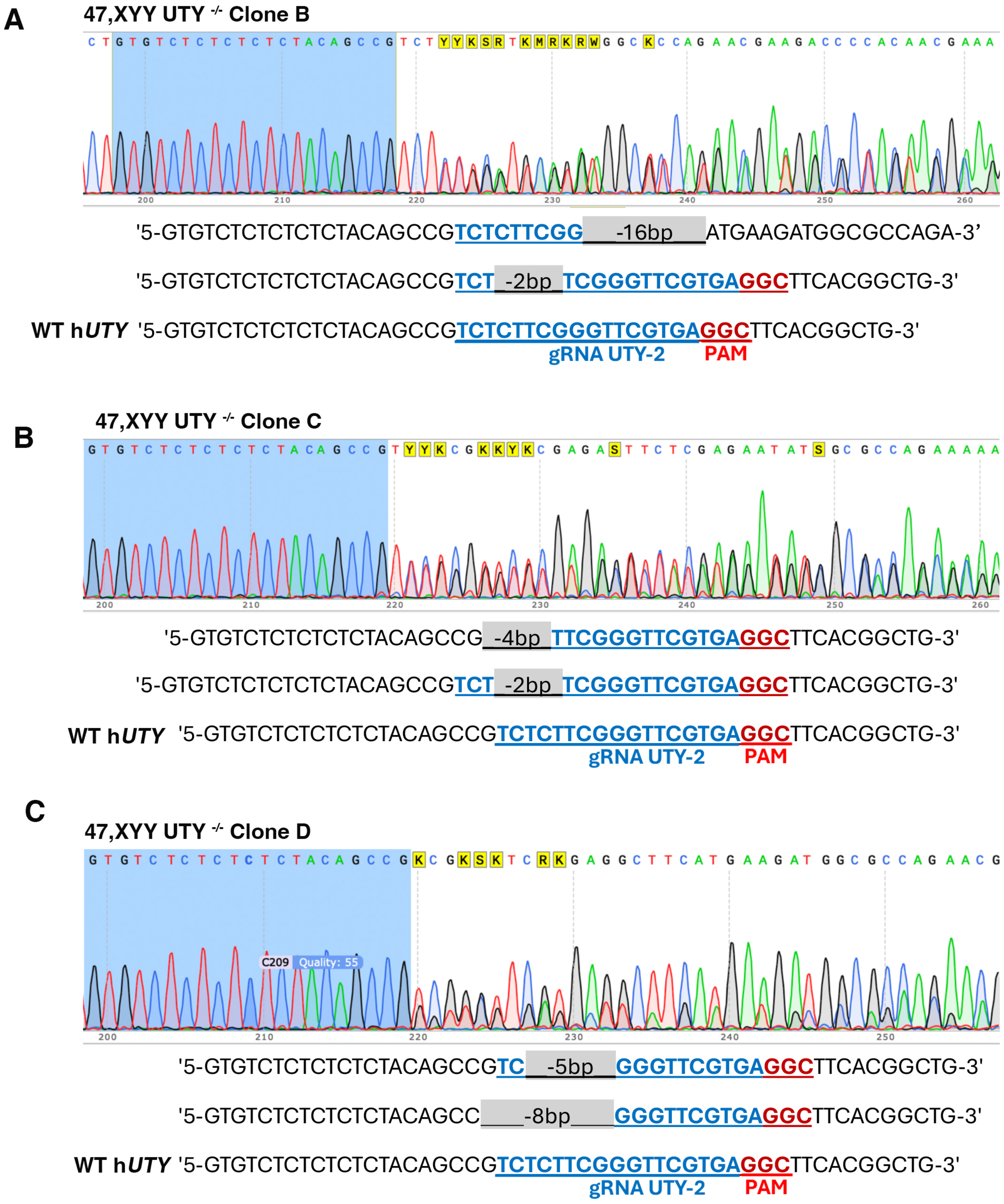

Supplemental_Fig_S15

Supplemental Fig S15: Generation of 47,XYY UTY Knock-out iPSCs.

A-C) Electropherograms of three independent JS-iPSC clones edited for *UTY* using gRNA UTY-2 to generate 47,XYY UTY $^{-/-}$ iPSCs.

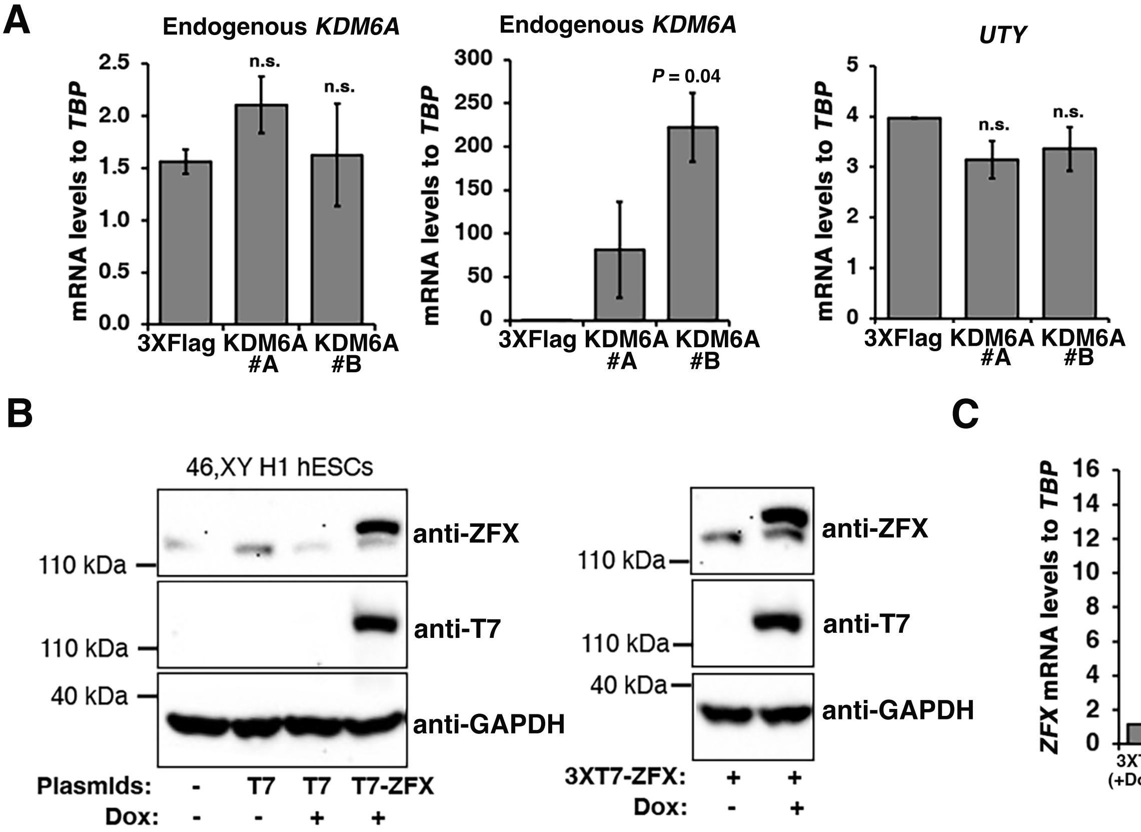

Supplemental_Fig_S16

Supplemental Fig S16: Overexpression of NPX-NPY gene pairs in H1 46,XY hESCs.

A) Taqman assay for endogenous *KDM6A* (left), exogenous gene expression by Real-time qPCR (middle), and mRNA levels of *UTY* in 46,XY hESCs stably integrating either empty 3X-Flag (control) or 3X-Flag-KDM6A vectors. Two independent hESC clones (KDM6A#A and KDM6A#B) have been tested. Bars are the average ± std of three independent experiments. Student's *t*-test, two-tailed distribution, two-sample unequal variance. *P* values are indicated. The significance is set at *P* value < 0.05. **B)** Left: Protein levels of ZFX in non-transfected, 3XT7 empty (T7) or 3XT7-ZFX inducible 46,XY H1 hESCs. Right: Inducible overexpression of ZFX protein in hESCs H1 treated for 48 hrs with 50 ng/ml Doxycycline (+Dox) or not treated (-Dox). **C)** Taqman assay assessing *ZFX* and *ZFY* expression levels in Doxycycline (+Dox) hESCs transfected with 3XT7 empty (T7) or 3XT7-ZFX plasmids. n.s = not significant. ***P* value < 0.005

A

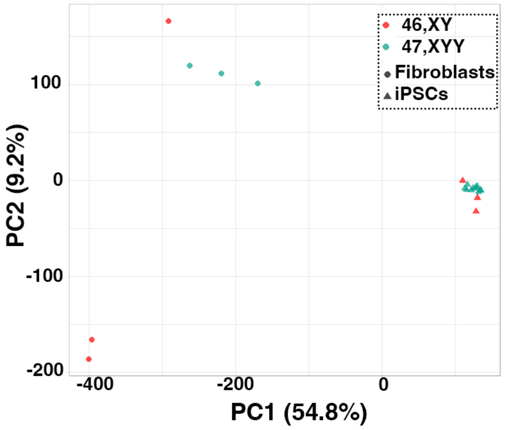

B

Cell type	Gene Location	Hyper_total	Hypo_total
Fibroblasts	Autosome	3384	691
Fibroblasts	X Chromosome	93	21
Fibroblasts	Y Chromosome	8	0
iPSCs	Autosome	28	31
iPSCs	X Chromosome	2	0
iPSCs	Y Chromosome	0	2

Supplemental_Fig_S17

Supplemental Fig S17: Methylation analysis in 47,XYY A) PCA of methylation sites in fibroblasts and iPSCs on the whole genome and **B)** Number, location, and direction of DMRs in fibroblasts and iPSCs.

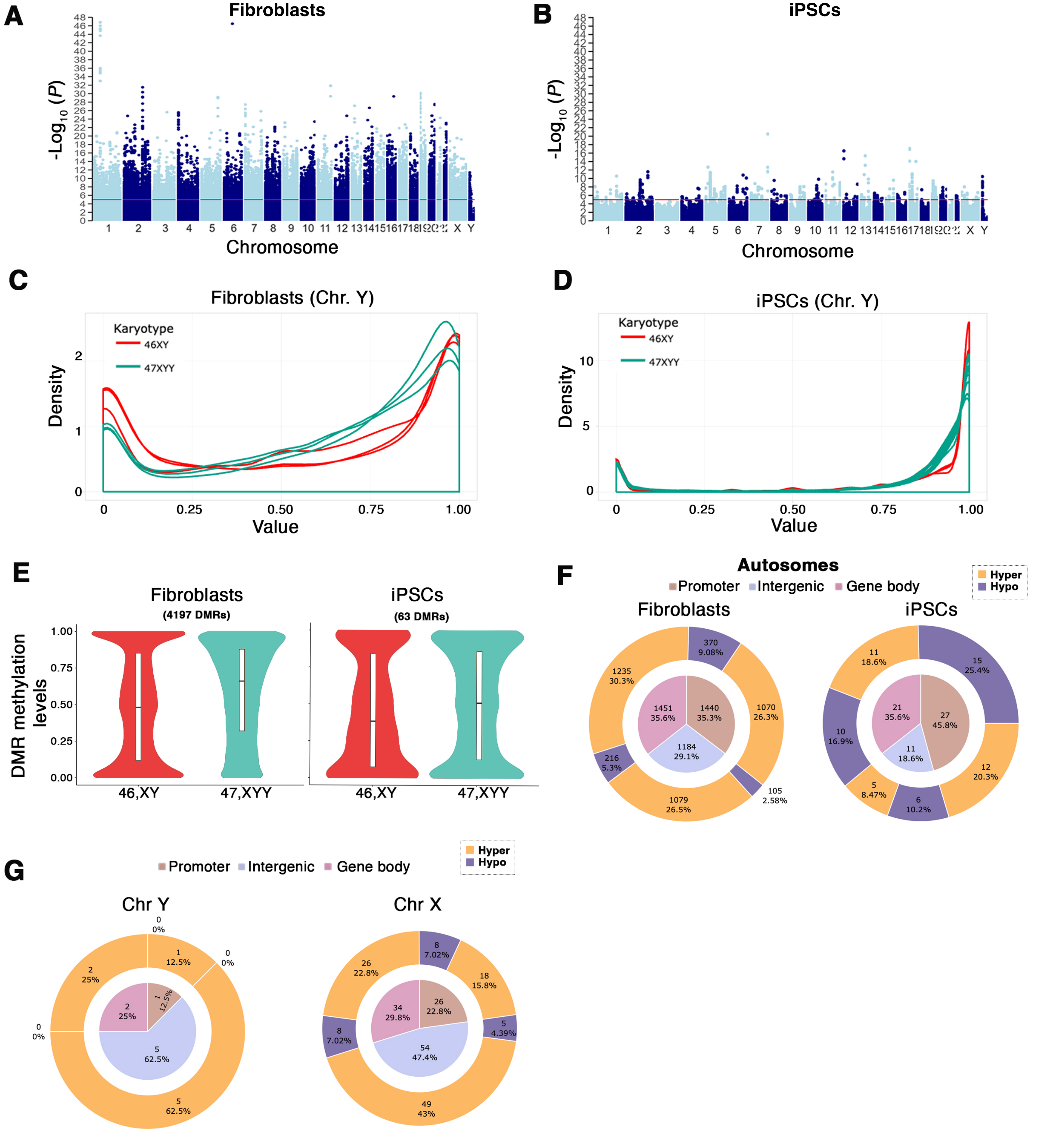

Supplemental_Fig_S18

Supplemental Fig S18: DNA Methylation profile of 47,XYY and 46,XY in fibroblasts and iPSCs. A-B) Manhattan Plots showing the differential methylation in CpG sites between 47,XYY and 46,XY in A) fibroblasts and B) iPSCs (above the red line, *P* value < 1×10^{-5}). Each point represents a single CpG site, y-axis shows negative $\log_{10}$ of associated *P* values. **C-D)** Density plots of the CpG methylation ratios in Y Chromosome in 47,XXY vs. 46,XY in fibroblasts (C) and (D) iPSCs. **E)** Violin distribution plot in DMR methylation levels in 47XYY vs. 46XY fibroblasts and iPSCs. The X-axis represents the comparison karyotype, and the Y-axis represents the methylation level value. The inner Box plot represents the ends of the first and third quartile, and the central slash represents the median. **F)** Nested pie chart of the distribution of autosomal DMRs in Fibroblast (left) and iPSCs (right). **G)** Nested pie chart of the distribution of DMRs in Y (left) and X (right) Chromosomes in fibroblasts. The Inner pie represents the proportion of DMR located in the gene promoter (brown), gene body (pink), and intergenic regions (blue). The Outer donut represents the proportion of the DMRs in the inner pie categories that are hypermethylated (orange) or hypomethylated (blue).

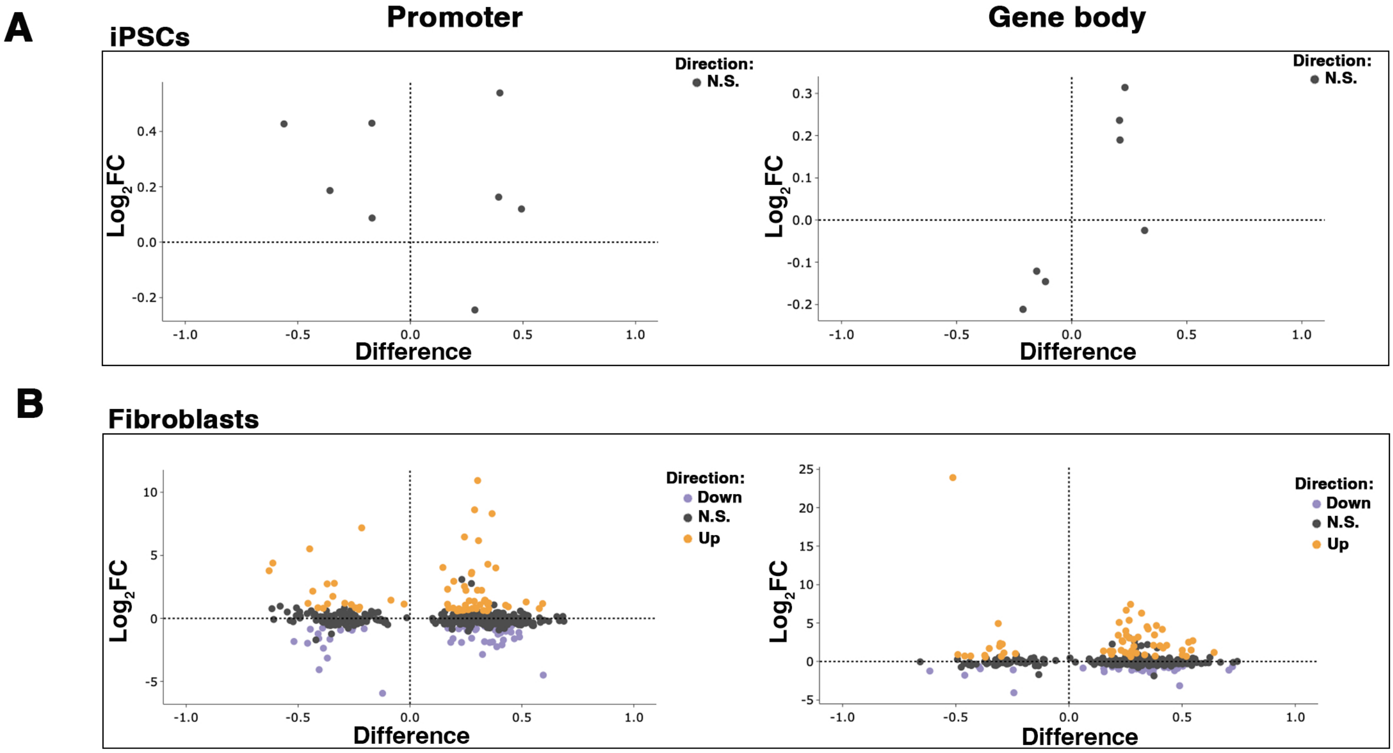

Supplemental_Fig_S19

Supplemental Fig S19: Methylation status of genes differentially expressed in fibroblasts and iPSCs. Scatter plot showing the correlation of the average methylation over DMRs located in the promoter and gene body with the gene expression (DESeq2 normalized data) in **A)** iPSCs and **B)** fibroblasts. Each dot represents a gene. Only genes with a coefficient of determination R^2 > |0.2| are shown. For genes containing more than 1 DMR in a specific region (promoter/gene body) the average methylation between the multiple DMRs was calculated.

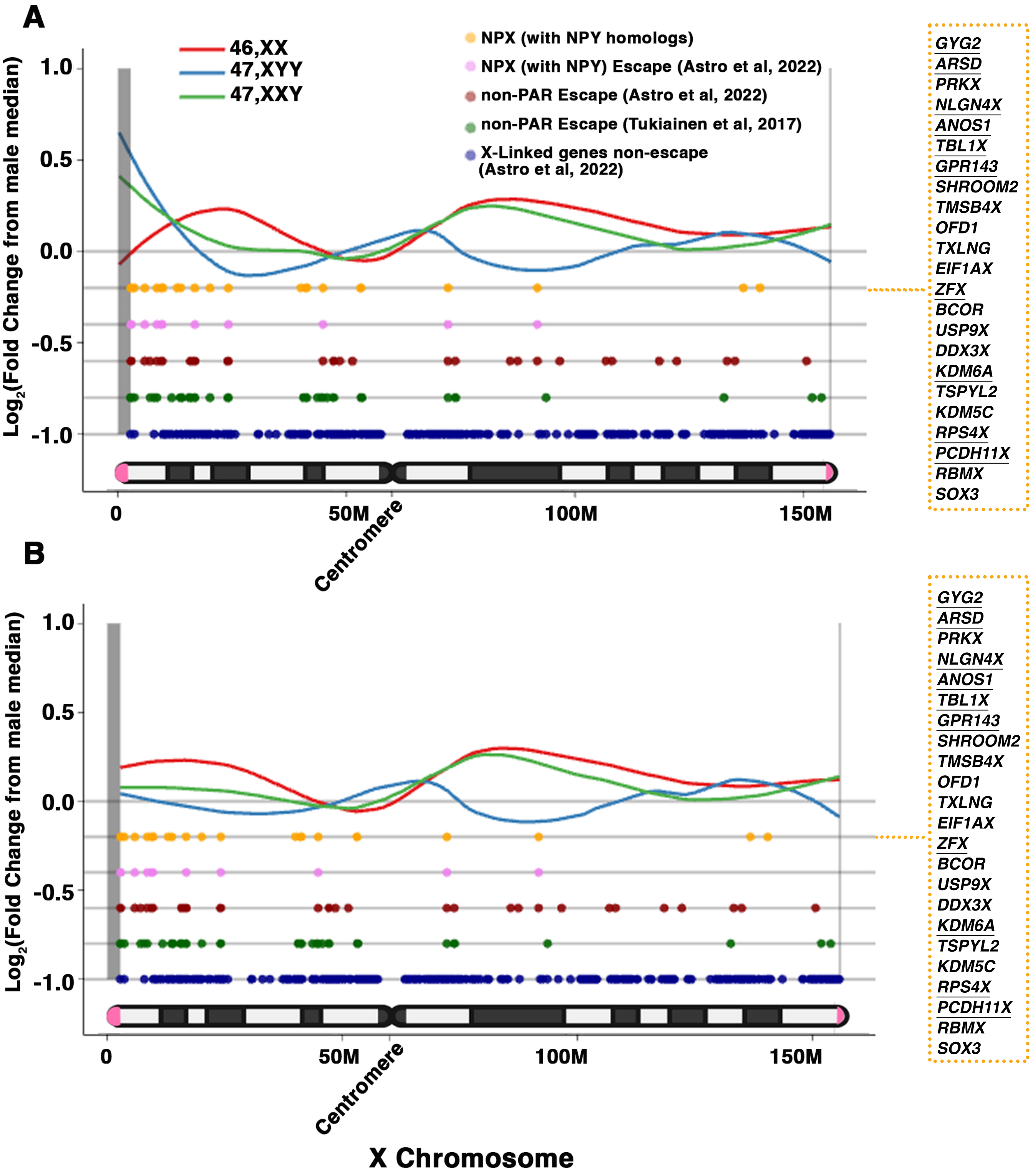

Supplemental_Fig_S20

Supplemental Fig S20: Moving expression average plot along the X Chromosome

A-B) Moving average line plot (loess fit, span 0.45) along the X Chromosome showing the Log_2 fold change from control 46,XY iPSCs. Gray vertical boxes indicate PAR1 and PAR2 regions. The gray horizontal line represents no theoretical deviations from control 46,XY samples. In **B)**, PAR1 genes are masked. The location of four gene categories is displayed: NPX genes with a NPY homolog (yellow dots); NPX genes escaping X inactivation with a NPY homolog (Astro et al., 2022) (pink dots); non-PAR escape genes (Tukiainen et al., 2017) (green dots); non escape X-linked genes (Astro et al., 2022) (blue dots). NPX genes with NPY homologs expressed in iPSCs are listed on the right. NPX genes escaping X inactivation are underlined.

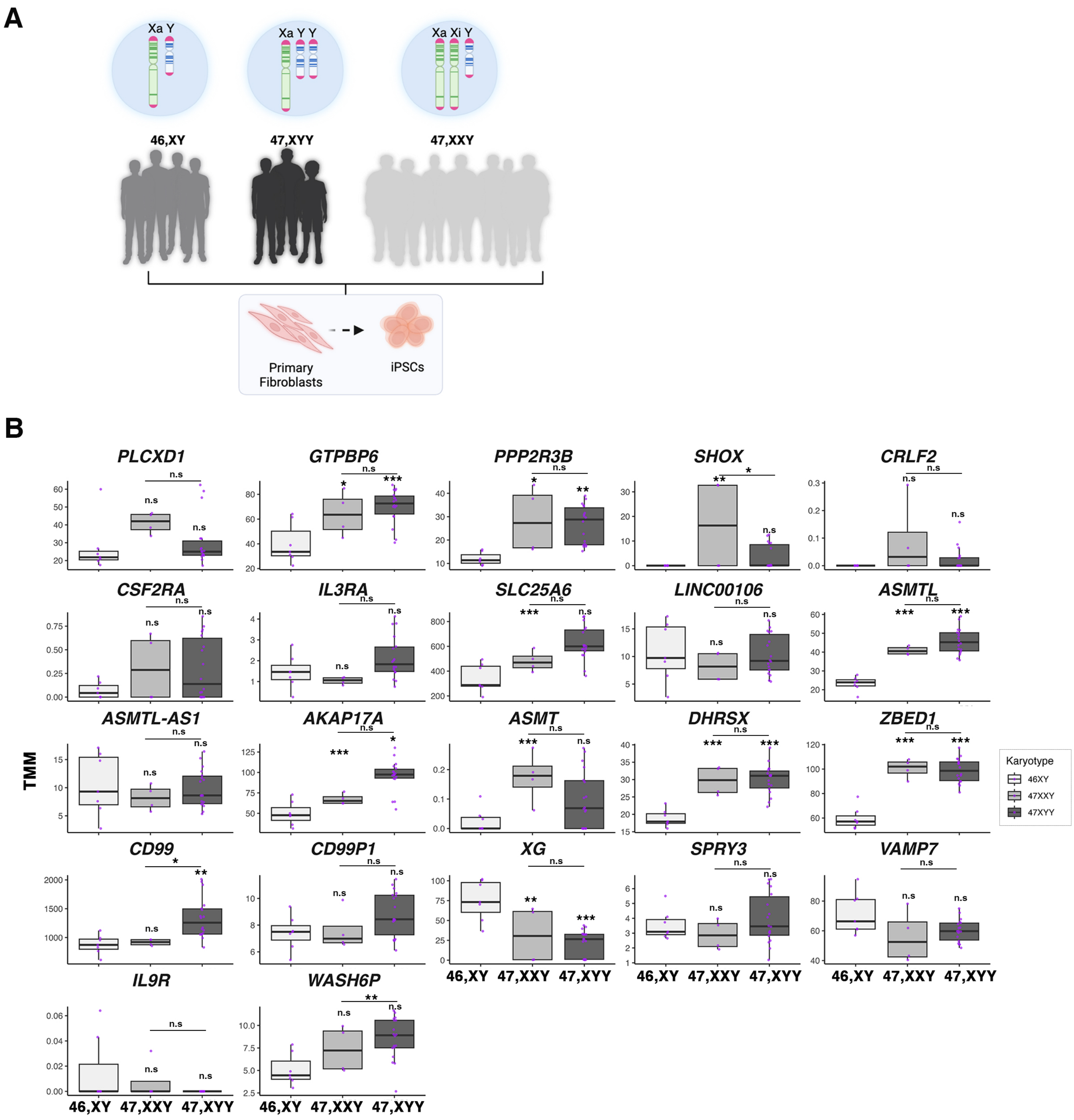

Supplemental_Fig_S21

Supplemental Fig S21: PAR gene expression in 47,XYY, 47,XXY, and 46,XY fibroblasts. A) Schematic of JS, KS, and control cohort of patients used in the study. **B)** Box plot of the TMM Normalized PAR gene expression in control 47,XYY, 47,XXY, and 46,XY fibroblasts. Each Purple dot represents an independent RNA sample. One-way ANOVA and post-hoc Tukey HSD analyses were used to test the significance between karyotypes. *P value < 0.05; **P value < 0.01; ***P value < 0.001.
